# Arrhythmic events and genetic variants as drivers for Takotsubo Syndrome: a patient-specific atrial stem-cell model

**DOI:** 10.1038/s41392-026-03000-z

**Published:** 2026-10-23

**Authors:** Charlotte Brand, Clarissa Zinke, Gideon Syed Ali, Daniela Hübscher, Karim Ajmail, Aylin Seedorf, Pauline Steinker, Franziska Treu, Philipp Jung, Hanna Eberl, Yun Li, Nataliya Dybkova, Lukas Feld, Jelena R. Ghadri, Christian Templin, Samuel Sossalla, Christoph Maack, Niels Voigt, Viacheslav O. Nikolaev, Bernd Wollnik, Katrin Streckfuss-Bömeke

**Affiliations:** 1 Clinic for Cardiology and Pneumology, University Medical Centre Göttingen, Göttingen, Germany; 2 Institute for Cardiomyopathies (ICH), University Clinic Heidelberg, Heidelberg, Germany; 3 DZHK (German Centre for Cardiovascular Research), partner site Lower Saxony, Göttingen, Germany; 4 Institute of Pharmacology and Toxicology, University of Würzburg, Würzburg, Germany; 5 Institute of Pharmacology and Toxicology, University Medical Centre Göttingen, Georg-August-University Göttingen, Göttingen, Germany; 6 Institute of Human Genetics, University Medical Centre Göttingen, Göttingen, Germany; 7 Centre for Molecular Cardiology, Schlieren Campus, University of Zurich, Zurich, Switzerland; 8 Internal Medicine B, University Medicine Greifswald, Greifswald, Germany; 9 Department of Cardiology, University Hospital Zurich, Zurich, Switzerland; 10 DZHK (German Centre for Cardiovascular Research), partner site North, Hamburg, Lübeck, Kiel, Greifswald, Germany; 11 Medical Clinic I, Cardiology and Angiology, Justus-Liebig-University, Gießen, Germany; 12 Department of Cardiology, Campus Kerckhoff, Bad Nauheim, Germany; 13 Cardio-Pulmonary Institute (CPI), Gießen, Germany; 14 Comprehensive Heart Failure Center (CHFC), University Clinic Würzburg, Würzburg, Germany; 15 Medical Clinic and Polyclinic I, University Clinic Würzburg, Würzburg, Germany; 16 Cluster of Excellence ‘Multiscale Bioimaging: From Molecular Machines to Networks of Excitable Cells’ (MBExC), University Medical Centre Göttingen, Göttingen, Germany; 17 Department of Pharmacology and Toxicology, European Center for Angioscience, University Heidelberg, Mannheim, Germany; 18 Institute for Experimental Cardiovascular Research, University Medical Centre Hamburg-Eppendorf, Hamburg, Germany; 19 German Centre for Child and Adolescent Health (DZKJ), partner site Göttingen, Göttingen, Germany

**Keywords:** Cardiology, Pluripotent stem cells

## Abstract

Takotsubo Syndrome (TTS) is an acute, potentially life-threatening condition often complicated by ventricular dysfunction and arrhythmias. Atrial fibrillation (AF) is common in TTS patients and associated with increased mortality. However, the arrhythmic mechanisms, genetic variability underlying TTS, and personalised therapy remain unclear. We generated induced pluripotent stem cell-derived atrial and ventricular cardiomyocytes (iPSC-CMs) from TTS patients and provoked TTS-like events by isoprenaline (Iso) stimulation. Compared with controls, *atrial* TTS iPSC-CMs exhibited enhanced diastolic sarcoplasmic reticulum (SR) Ca²⁺ leak and increased arrhythmogenic potential following Iso exposure. Iso-induced stress further increased systolic Ca²⁺ transient amplitude and reduced phosphodiesterase 4 (PDE4) activity in TTS iPSC-CMs. Under baseline conditions, atrial TTS iPSC-CMs demonstrated unchanged Ca^2+^ transient amplitude, reduced diastolic Ca^2+^, elevated SERCA2 activity with faster Ca²⁺ decay compared to controls, which could be explained by increased phosphorylation of PKA-specific phospholamban S16. In contrast, *ventricular* TTS-iPSC-CM showed increased L-Type Ca^2+^ current density, but reduced Ca^2+^ transient amplitude with increased resting Ca^2+^ leak and reduced PDE4 activity post-Iso-exposure. In atrial TTS iPSC-CMs, the PDE4 activator MR-L2 restored PDE4 activity, normalised several defects in Ca²⁺ handling and prevented arrhythmias more efficiently than the β-blocker metoprolol. Genetic analyses identified variants in the Ca²⁺ handling genes *AHNAK* and *CASQ2* in TTS. Using CRISPR/Cas9, we generated isogenic rescue and mutation-insertion lines, confirming a causal role of p.P5390T in catecholamine-induced arrhythmogenic phenotypes in atrial TTS iPSC-CMs. Our findings support a mechanistic link between genetic predisposition to an excessive catecholaminergic stress response, dysregulated PDE/cAMP signalling, and atrial arrhythmogenesis in TTS-CMs, identifying PDE4 as a potential therapeutic target in this critical condition.

## Introduction

Takotsubo Syndrome (TTS), also known as “Broken Heart Syndrome” or stress-induced cardiomyopathy, is an often-transient cardiac disorder characterised by acute left ventricular (LV) dysfunction in the absence of significant coronary artery obstruction.^1^ Although TTS was initially considered a benign condition, more than 20% of patients experience severe in-hospital complications at rates comparable to or exceeding those observed in patients with acute coronary syndrome.^2^ TTS has a broad spectrum of clinical manifestations, which often include arrhythmias that significantly impact patient outcomes.^3^ In 7–25% of TTS cases, atrial fibrillation (AF) occurs either during the acute phase or after recovery from TTS.^4–6^ Furthermore, AF is an independent risk factor for mortality, which more than doubles the risk in long-term follow-up studies of patients with TTS.^7^ However, while previous research focused primarily on the mechanisms of LV dysfunction, little is known about the pathophysiology of AF in TTS.

Currently, there is no specific treatment strategy for TTS, and patients are treated according to the heart failure (HF) phenotype that emerges, which is commonly HF with reduced ejection fraction (HFrEF). In patients with HFrEF, excessive activation of the sympathetic nervous system predicts adverse outcomes^8^ due to chronic activation of cardiac β-adrenergic receptors (β-ARs), which leads to desensitisation and uncoupling of β-ARs as well as dysregulation of cytosolic Ca^2+^ handling, arrhythmias, cardiac hypertrophy and cell death.^9^ Therefore, β-AR blockade with β-blockers is one of the four main pillars of HFrEF therapy and substantially improves prognosis.^10^ In patients with TTS, however, the use of β-blockers remains controversial,^11,12^ although treatment at discharge from hospital was associated with a 29% lower mortality in long-term follow-up, without affecting TTS recurrence or recovery of LV function.^13^ Management of arrhythmias in TTS remains complex and nuanced. Amiodarone, the generally most efficient antiarrhythmic and yet safe drug, may prolong the QTc interval by blocking potassium channels and interfering with β-ARs, Ca^2+^- and Na^+^-channels, potentially inducing arrhythmias and worsening TTS.^14^ Cases of Amiodarone-induced TTS, probably due to iatrogenic hyperthyroidism, have been described,^15^ underlining that safer, more targeted therapies are needed, particularly for arrhythmias in TTS.^16^

Although the exact pathophysiological mechanisms are poorly understood, TTS appears to result from a complex interplay of multiple factors.^11^ Catecholamine overstimulation has been identified as a significant trigger of cardiac dysfunction in TTS patients.^17–20^ Catecholamines initiate the elevation of cyclic adenosine monophosphate (cAMP) through β-AR stimulation.^21^ Elevated cAMP in human atrial myocytes enhances cardiac function via a protein kinase A (PKA) dependent ryanodine receptor 2 (RYR2) activation, increasing its open probability and provoking spontaneous sarcoplasmic reticulum (SR) Ca²⁺ release events, so-called Ca^2+^ “sparks”, which can trigger arrhythmias through delayed afterdepolarisations (DADs) of the membrane potential.^22–24^ To prevent overstimulation, cAMP levels are degraded by various phosphodiesterases (PDEs), and in the human heart, PDE3 and PDE4 are the most relevant.^25^ Inhibition of PDE4 disrupts this balance, elevating cAMP levels, triggering Ca^2+^ “sparks” through RyR2 phosphorylation, and increasing SR Ca^2+^ load.^26,27^ Patients with AF exhibit reduced PDE4 activity, increasing their susceptibility to arrhythmias.^22^ Therefore, PDE4 plays a protective role in maintaining cardiac rhythm, highlighting its significance in AF pathophysiology.^22,23^

Recently, a novel drug for the treatment of autosomal dominant polycystic kidney disease (ADPKD), a disease characterised by PDE4 downregulation, has shown promising effects in animal and in vitro models by specifically activating PDE4 and thereby lowering cytosolic cAMP.^28^ Furthermore, we previously identified mutations in cardiac-relevant genes in TTS patients.^18^ Calsequestrin 2 (CASQ2) functions as a Ca^2+^ buffer within the SR.^29^ While various mutations in *CASQ2* have been associated with catecholaminergic polymorphic ventricular tachycardia (CPVT),^30^ the F189L variant was also found in patients with TTS.^31,32^ The giant protein AHNAK (also known as desmoyokin) regulates the cardiac L-type Ca^2+^ channel current (*I*_Ca,L_) by controlling the availability of its β2 subunit.^33^ Thus far, the impact of *AHNAK* variants on the development of TTS-related arrhythmias remains unknown.

Induced pluripotent stem cells (iPSCs) represent a groundbreaking advancement in biomedical research, enabling the modelling of patient-specific diseases and the exploration of therapeutic options.^34^ Their capacity to differentiate into diverse cell types makes them especially valuable for studying cardiac diseases, reliably reflecting disease phenotypes in vitro.^18,35^ Previously, we and others have shown that iPSC-derived atrial and ventricular cardiomyocytes (CMs) provide a genetically adaptable model that enables precise functional studies of proarrhythmic mechanisms, including late sodium currents and Ca^2+^ handling.^36–38^ Their efficiency and ability to consistently reproduce disease-relevant phenotypes make them well-suited for large-scale in vitro modelling and targeted electrophysiological and pharmacological investigations.^39,40^ The gene-modifying tool clustered regularly interspaced short palindromic repeats (CRISPR) and CRISPR-associated protein 9 (CRISPR-Cas9) allows for the targeting of specific mutations and the analysis of their impact on cellular and molecular function.^41^

Despite the clinical significance of atrial arrhythmias in TTS, the underlying cellular mechanisms and genetic determinants remain poorly understood, and evidence-based therapeutic strategies are lacking. Here, by integrating patient-specific disease modelling, gene-editing approaches, and mechanistic analyses, we identify *AHNAK* variants as potential drivers of atrial arrhythmogenesis, providing new insights into TTS disease pathophysiology and a rationale for the development of personalised therapeutic strategies.

## Results

### Clinical presentation of TTS patient

In this study, atrial iPSC-CMs from a TTS patient were used to investigate disease-related mechanisms in TTS. The patient (registered in the International Takotsubo Registry (NCT01947621)) was a 67-year-old postmenopausal woman who experienced a single TTS episode characterised by classical apical ballooning. This event was associated with physical neurological stressors including acute stroke and acute seizure, whereas no emotional triggers were identified. During the acute presentation, the TTS patient did not report chest pain or dyspnoea. Initial echocardiography revealed an apical TTS type with a reduced LV ejection fraction (LVEF) of 35%. Subsequent coronary angiography demonstrated an elevated LV end-diastolic pressure (LVEDP) of 24 mmHg and confirmed the reduced LVEF (30%), while obstructive coronary artery disease (CAD) could be ruled out. No relevant arrhythmias were detected on admission and during early follow-up (days 1-3 and days 4-7). Initial ECG on admission showed sinus rhythm, ST elevation and a QTc interval of 435 ms. After 1-3 days, ST depression, T-wave inversion, and QTc prolongation (493 ms) were noted. ST depression and T-wave inversion persisted after 4-7 days, whereas the QTc interval decreased to 455 ms. Three days after the initial TTS event, the patient developed a subdural hygroma and hypoliquorrhoe syndrome. Known comorbidities included arterial hypertension and hypercholesterolaemia. On admission, the patient was treated with an angiotensin receptor blocker (ARB). At discharge, medical therapy consisted of an ARB, a β-blocker and a benzodiazepine. An overview of the key clinical findings is provided in Table 1.

**Table 1:** Clinical findings of the study’s TTS patient

Patient	TTS 5
**Sex**	Female
**Age at TTS event**	67 years (postmenopause)
**TTS type**	Apical ballooning
**Trigger**	Neurological disorder: seizure with vigilance, acute stroke
**LVEF on admission**	Echocardiography: 35%; Catheter lab: 30%
**Heart rate on admission**	75 bpm
**ECG findings on admission**	Sinus rhythm, ST elevation, QTc: 435 ms
**ECG findings after 1-3 days**	Sinus rhythm, ST depression, T inversion, QTc: 493 ms
**ECG findings after 4-7 days**	Sinus rhythm, ST depression, T inversion, QTc: 455 ms
**Coronary angiography findings**	no obstructive CAD
**Comorbidities**	Arterial hypertension, Hypercholesterolaemia
**Discharge medication**	β-blocker, AT_1_ -blocker, Benzodiazepine

All iPSC lines used in this study were analysed thoroughly before differentiation into iPSC-CMs. The CTRL and the TTS patient (TTS patient 5) cell lines were described previously by Borchert et al.^18^ iPSCs were differentiated into atrial CMs. All atrial iPSC-CMs presented with the characteristic elongated morphology and a regular sarcomeric striated pattern, visualised via immunofluorescence staining of the cardiac proteins α-actinin and titin (Supplementary Fig. 1a). Cardiac differentiation efficiency was analysed via flow cytometry. 87.1% ±5.5% of all generated cells were identified as positive for cTNT (Supplementary Fig. 1b). Combined immunofluorescence staining of the atrial and ventricular isoforms MLC2a/v showed that 90.9% ± 6.3% of the analysed cells were positive for MLC2a and negative for MLC2v (MLC2a+/MLC2v-) (Supplementary Fig. 1c, d). Furthermore, successful atrial differentiation was confirmed at the mRNA level, showing high expression of the atrial marker Nuclear Receptor Subfamily 2 Group F Member 2 (NR2F2) and low expression of *MLC2v* (Supplementary Fig. 1e, f). No difference regarding atrial differentiation was observed between the analysed cell lines.

### Atrial TTS cells exhibit an increased diastolic SR Ca^2+^ leak and a high number of arrhythmic events

**Fig. 1:**
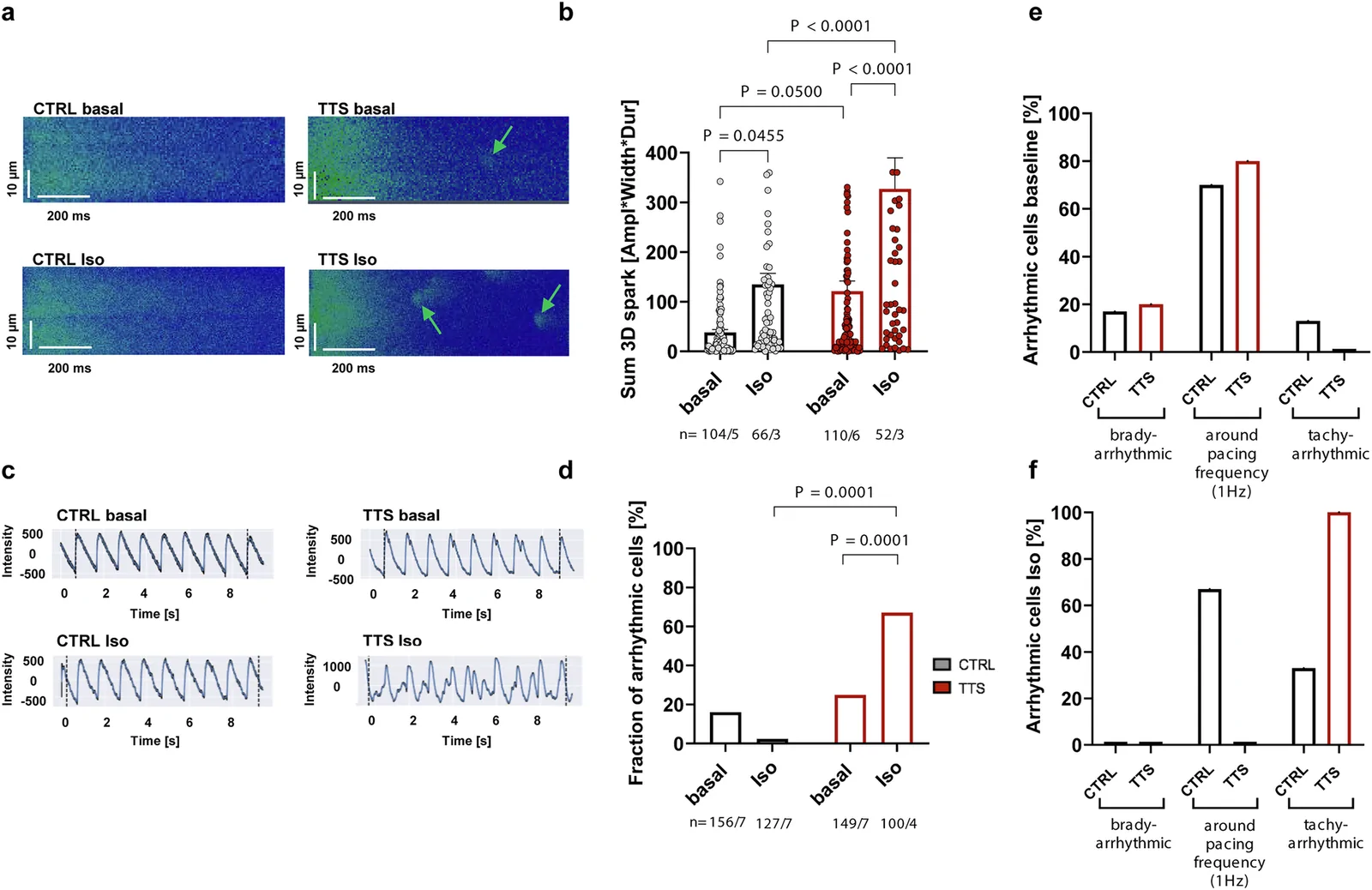
Diastolic SR Ca^2+^ leak and and arrhythmias in atrial TTS iPSC-CMs. **a** Exemplary line scans of CTRL iPSC-CMs and TTS iPSC-CMs under baseline conditions and after Iso treatment (100 nM) (green: cytosolic Ca^2+^, blue: background, arrows: Ca^2+^ sparks; scale bar: height = 10 µm, length = 200 ms). **b** 3D sum of all identified Ca^2+^ sparks (baseline conditions: TTS cells (*n* = 110 cells/6 diffs), CTRL cells (*n* = 104/5); after 100 nM Iso treatment: TTS cells (*n* = 52/3), CTRL cells (*n* = 66/3). Two-way ANOVA analysis with Post hoc tests used the Tukey correction for multiple comparisons. Data are presented as mean values ± SEM. **c** Exemplary arrhythmia analysis of a line scan of a CTRL cell and a TTS cell under baseline conditions and after application of 100 nM Iso. **d** Fraction of arrhythmic TTS iPSC CMs (baseline condition: *n* = 149/7; 100 nM Iso: *n* = 100/4) and CTRL iPSC CMs (baseline condition: *n* = 156/7; 100 nM Iso: *n* = 127/7). Fisher’s exact test analysis used for comparison of two groups: CTRL versus TTS (baseline), TTS baseline versus TTS Iso. **e**, **f** Classification of arrhythmic cells into bradyarrhythmic, normofrequent (around pacing frequency), and tachyarrhythmic subgroups. **a**, **b**, **e**, **f** Cells were paced at 1 Hz. **a**, **b** Confocal microscopy (Fluo 4-AM); **d**–**f** Transients and analysis of arrhythmic cells based on confocal microscopy line scans (Fluo 4-AM)

We hypothesised that atrial TTS iPSC-CMs exhibit increased arrhythmogenic response under severe stress conditions. Since spontaneous SR Ca^2+^ release events can serve as potential triggers for arrhythmias such as AF,^42^ we performed confocal microscopy (Fluo 4-AM) and calculated the diastolic SR Ca^2+^ leak by multiplying the amplitude, width, and duration of Ca^2+^ sparks. To mimic TTS stress conditions, we used isoprenaline (Iso) to stimulate β-ARs in all cells. We observed increased Ca^2+^ sparks and thereby SR Ca^2+^ leak in atrial TTS iPSC-CMs compared to the CTRL cell line under all conditions. The effect was more pronounced upon Iso stimulation, resulting in a 2.4-fold increase in diastolic Ca^2+^ leak in TTS cells compared to CTRL cells (Fig. 1a, b).

To detect and quantify arrhythmias, confocal microscopy scans (Fluo 4-AM) were analysed using *Arrhythpy*, an automated arrhythmia quantification tool.^43^ The distinction between rhythmic and arrhythmic recordings was based on deviations of the measured beating frequency from the stimulation frequency (1 Hz) and on the correlation between individual peaks. Exemplary transients are shown in Fig. 1c and further classified in Fig. 1d. In response to catecholamine stress, the increased Ca^2+^ leak promoted the occurrence of arrhythmic events in 67% of TTS cells. In contrast, CTRL cells continued to exhibit rhythmic beating under the same conditions (Fig. 1c, d). We further characterised the types of arrhythmic events at baseline (Fig. 1e) and under Iso stress (Fig. 1f). At baseline, 70% of arrhythmic CTRL cells beat near the pacing frequency (PF, 1 Hz), while 17% were bradyarrhythmic and 13% tachyarrhythmic. In TTS cells, 80% beat near PF and 20% were bradyarrhythmic. Iso markedly shifted these profiles: in CTRL, 67% remained near PF, and 33% became tachyarrhythmic, whereas all arrhythmic TTS cells (100%) exhibited tachyarrhythmic behaviour.

### Dysfunctional Ca^2+^ homoeostasis and disturbed electrophysiological behaviour in atrial TTS iPSC-CMs

**Fig. 2:**
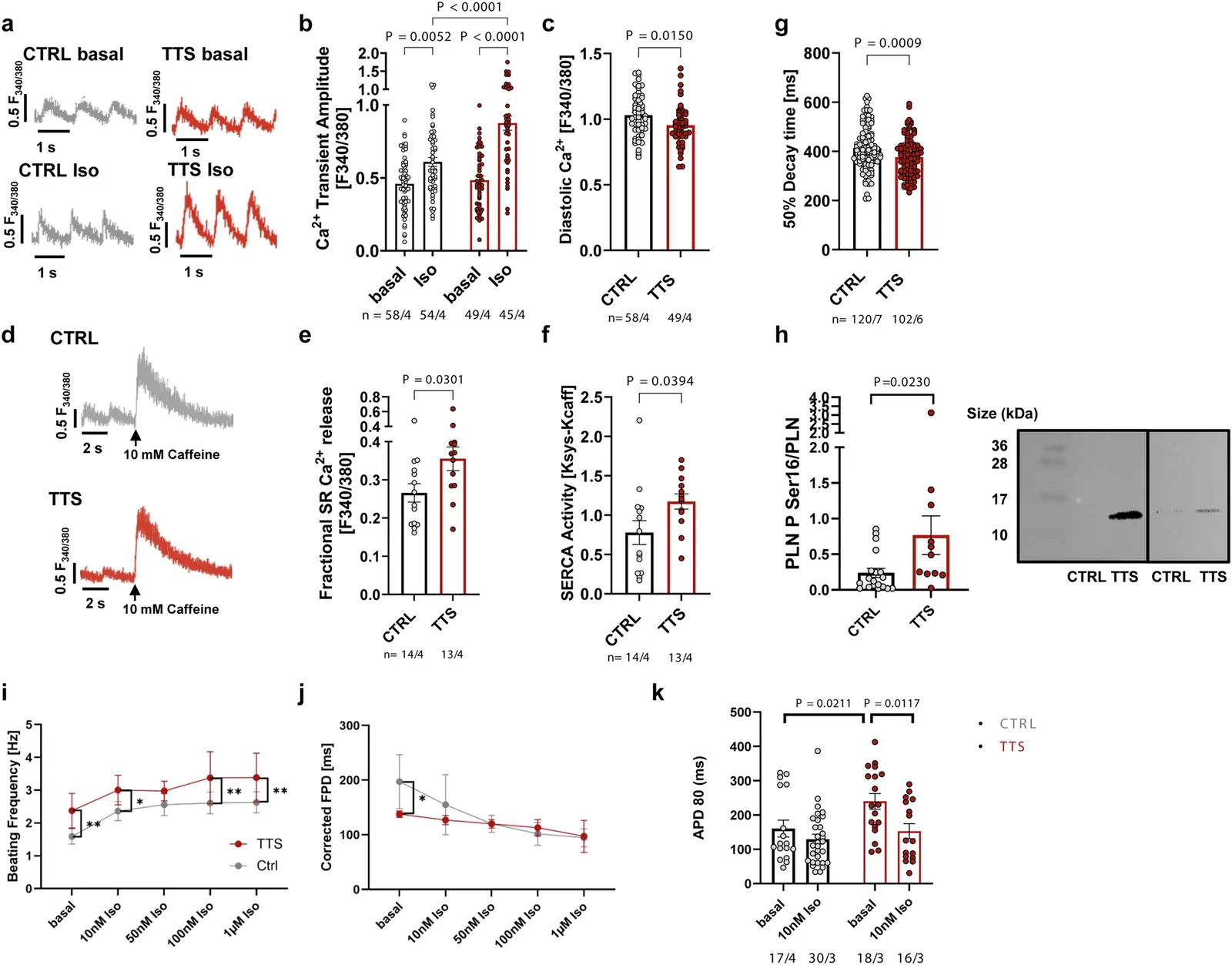
Dysfunctional Ca^2+^ homoeostasis in atrial TTS iPSC-CMs. **a** Exemplary Ca^2+^ transients of CTRL (grey) and TTS iPSC-CMs (red) under baseline conditions and after Iso application (10 nM) (scale bar: height = 0.5F_340/380_, length = 1 s). **b** Ca^2+^ transient amplitude in atrial TTS iPSC-CMs (*n* = 45 cells/4 diffs) and CTRL cells (*n* = 54/4) after Iso treatment (10 nM) and at baseline level (TTS: *n* = 49/4; CTRL: 58/4) (Two-way ANOVA with Post hoc Tukey test). **c** Diastolic Ca^2+^ levels in TTS iPSC-CMs (*n* = 49 cells/4 diffs) and CTRL iPSC-CMs (*n* = 58/4) (unpaired t-test). **d** Exemplary Ca^2+^ transients after Caffeine application (10 mM) of the CTRL cell line (grey) and the TTS cell line (red) (scale bar: height = 0.5F_340/380_, length = 2 s). **e** Fractional SR Ca^2+^ release in TTS iPSC-CMs (*n* = 13 cells/4 diffs) and CTRL iPSC-CMs (*n* = 14/4). **f** SERCA2a activity (K_sys_-K_caff_) in TTS iPSC-CMs (*n* = 13 cells/4 diffs) and CTRL iPSC-CMs (*n* = 14/4) (unpaired t-test). **g** 50% relaxation time in TTS cells (*n* = 102 cells/6 diffs) and CTRL cells (*n* = 120/7) (Mann-Whitney U test). **h** Western blot analysis for PLN phosphorylation (Ser16) in TTS cells (*n* = 11 diffs and CTRL cells (*n* = 19 diffs) under baseline conditions (unpaired t-test). **i**–**k** Electrophysiological measurements of CTRL and TTS iPSC-CMs in a syncytium (MEA) or at a single-cell level (patch clamp) under baseline and Iso-treated conditions. **i** Beating frequency in TTS cells (*n* = 5) and CTRL cells (*n* = 6) at baseline and after subsequent treatment with increasing concentrations of Iso (10 nM, 50 nM, 100 nM, 1 µM) (Two-way ANOVA with Post hoc Tukey test). **j** Fridericia corrected FPD under baseline conditions and after subsequent treatment with increasing concentrations of Iso (10 nM, 50 nM, 100 nM, 1 µM) in TTS cells (*n* = 5) and CTRL cells (*n* = 6) (Two-way ANOVA with Post hoc Tukey test). **k** APD under baseline conditions and upon Iso treatment (baseline condition: *n* = 18/3; 10 nM Iso: *n* = 16/3) CTRL (baseline condition: *n* = 17/4; 10 nM Iso: *n* = 30/3) (Two-Way ANOVA with Sidak’s multiple comparison test). **a**–**f** Epifluorescence microscopy (Fura 2-AM); **g** Confocal microscopy (Fluo 4-AM); **h** Western blot analysis; **i**, **j** MEA measurements; **k** Patch clamp analysis. **a**–**c** and **g** Cells were paced at 1 Hz. **d**–**f** Cells were paced at 0.5 Hz. All Data are presented as mean values ± SEM

We previously revealed that AF can cause distinct functional and molecular remodelling of the human LV.^44,45^ To gain further insight into the underlying pathophysiological mechanisms in TTS during AF, we investigated Ca^2+^ handling in atrial TTS iPSC-CMs using epifluorescence microscopy (Fura 2-AM). Iso treatment increased Ca^2+^ transient amplitude in both atrial CTRL and TTS iPSC-CMs compared to baseline, but with a more pronounced increase in TTS cells than in CTRL cells (Fig. 2a, b). Moreover, baseline diastolic Ca^2+^ levels were modestly decreased in TTS cells compared to CTRL cells (Fig. 2c). Depleting the SR of its stored Ca^2+^ through caffeine application (Fig. 2d) resulted in elevated fractional Ca^2+^ release from the SR (Fig. 2e) and enhanced SERCA2a activity (Fig. 2f) in TTS iPSC-CMs compared to CTRL iPSC-CMs.

Fluo-4 AM confocal microscopy showed a shorter time to 50% decay of Ca^2+^ in TTS cells than in CTRL cells (Fig. 2g), which could be traced to increased PKA-mediated phosphorylation of phospholamban (PLN) at S16 in TTS iPSC-CMs compared with CTRL (Fig. 2h). However, cAMP did not differ between TTS and CTRL iPSC-CMs under baseline conditions (Supplementary Fig. 2a). Taken together, the reduced diastolic Ca^2+^ level in TTS iPSC-CMs could be explained by enhanced SERCA2a activity that mediate a faster cytosolic Ca^2+^. To analyse if electrophysiological changes contribute to arrhythmia generation, we performed Multi-Electrode Array (MEA) measurements and found an increased spontaneous beating frequency in TTS cells compared to CTRL cells, both under baseline conditions and upon Iso stress (Fig. 2i). Consistent with the higher beating frequency, MEA further revealed a shorter corrected field potential duration (FPD) in TTS iPSC-CM syncitia at baseline (Fig. 2j), whereas single TTS iPSC-CMs showed a prolonged action potential duration (APD) relative to CTRL cells (Fig. 2k).

In conclusion, the phenotype of EC coupling in atrial TTS iPSC-CMs includes increased Ca^2+^ transient amplitudes and accelerated Ca^2+^ decay, accompanied by altered electrophysiological parameters.

### Decreased PDE4 activity upon catecholamine stress in atrial TTS iPSC-CMs

**Fig. 3:**
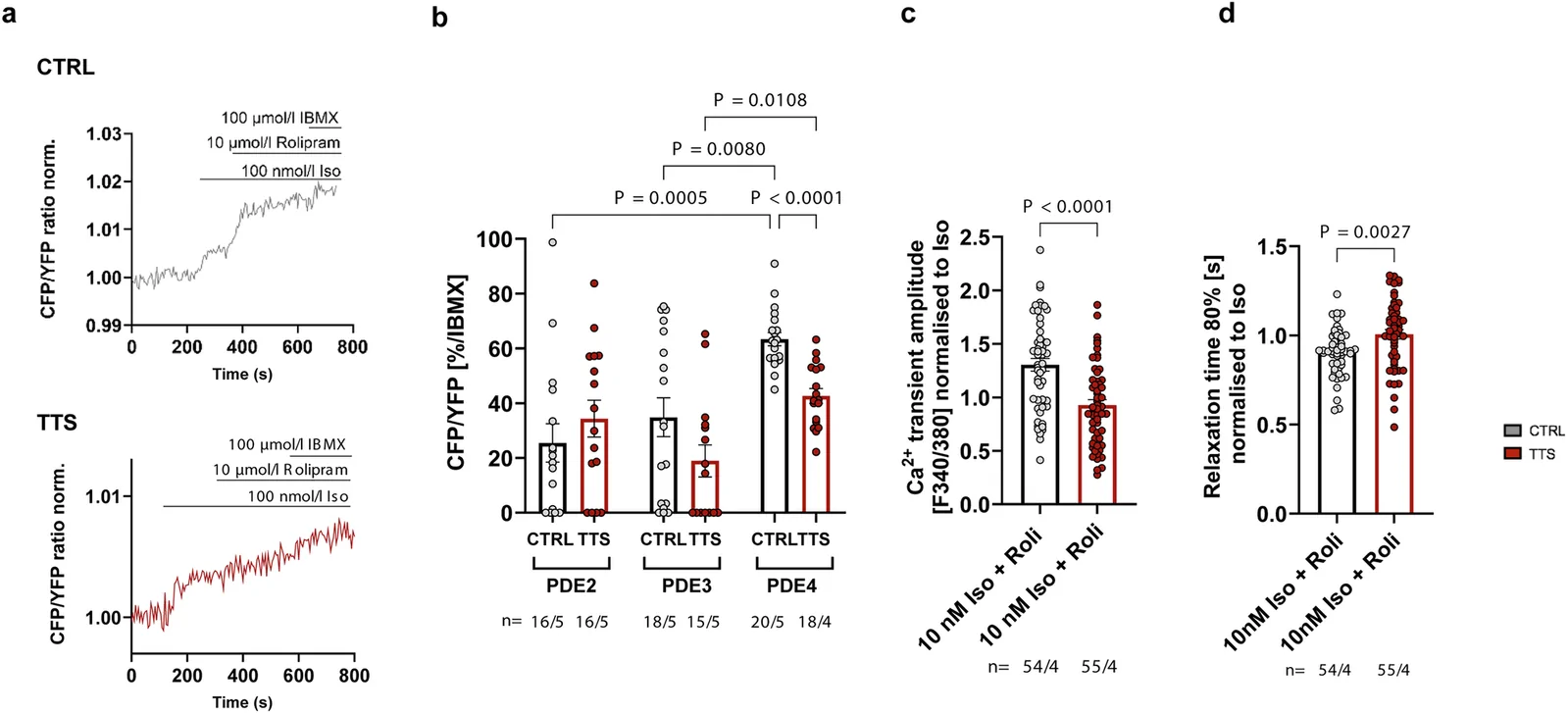
PDE activities in atrial TTS iPSC-CMs. **a** Representative CFP/YFP traces for CTRL- and TTS iPSC-CMs. **b** PDE2, PDE3, and PDE4 activity (as measured using an EPAC1-based FRET sensor for cAMP) after Iso treatment (100 nM) in TTS cells (PDE2: *n* = 16 cells/5 diffs, PDE3: *n* = 15/5, PDE4: *n* = 18/4) and CTRL cells (PDE2: *n* = 16/5, PDE3: *n* = 18/5, PDE4: *n* = 20/5) (Two Way ANOVA with Post hoc Tukey test). Shown are cAMP responses to individual PDE inhibitors as a % of maximal response evoked by 100 µM of the pan-PDE inhibitor IBMX. **c** Roli-induced (10 µM) increase in Ca^2+^ transient amplitude (normalised to Iso) in Iso-treated (10 nM) TTS cells (*n* = 55/4) and CTRL cells (*n* = 54/4) (unpaired t-test). **d** Roli-induced acceleration in 80% relaxation time in Iso-treated (10 nM) TTS iPSC-CMs (*n* = 55/4) and CTRL iPSC-CMs (*n* = 54/4) (unpaired t-test). **a**, **b** EPAC1-based FRET measurements; **c**, **d** Epifluorescence microscopy (Fura 2-AM), cells paced at 1 Hz. All Data are presented as mean values ± SEM

Since PDE4 activity is reduced in human atrial CMs from AF patients,^22^ we hypothesised that PDEs contribute to arrhythmogenesis during TTS development via cAMP regulation. Therefore, we studied PDE activities by transducing iPSC-CMs with an adenoviral construct expressing the Förster resonance energy transfer (FRET)-based cAMP sensor Epac1-camps under the control of the cytomegalovirus promoter.^46^ FRET measurements were performed by using PDE inhibitors of PDE2, 3, and 4 (100 nM Bay, 10 µM Cilostamid, 10 µM Rolipram (Roli)) (Fig. 3a). We did not observe any significant differences in basal PDE activities among the different isoforms (Supplementary Fig. 2b). After Iso treatment, the strongest control over cAMP was exerted by PDE4 compared to PDE2 and PDE3 (Fig. 3a, b). Under these conditions, TTS cells exhibited decreased PDE4 activity compared to CTRL cells, as evidenced by a lower Roli- (PDE4 inhibitor)-induced increase in cytosolic cAMP levels (Fig. 3b).

To test the functional consequences of lowered PDE4 activity in atrial TTS iPSC-CMs, epifluorescence microscopy (Fura 2-AM) revealed that PDE4-inhibition increased the amplitude of Iso-stimulated cytosolic Ca^2+^ transients in CTRL, but not more in TTS cells (Fig. 3c, Supplementary Fig. 2c). Accordingly, PDE4-inhibition accelerated diastolic Ca^2+^ decay only in Iso-stimulated CTRL, but not in TTS cardiac myocytes (Fig. 3d, Supplementary Fig. 2d). In conclusion, the supranormal β-adrenergic response of cytosolic Ca^2+^ handling (Fig. 2) is caused by dampened PDE4-related cAMP hydrolysis in TTS (Fig. 3).

### Dysfunctional Ca^2+^ homoeostasis and contractility in ventricular TTS iPSC-vCMs differ from the atrial phenotype

**Fig. 4:**
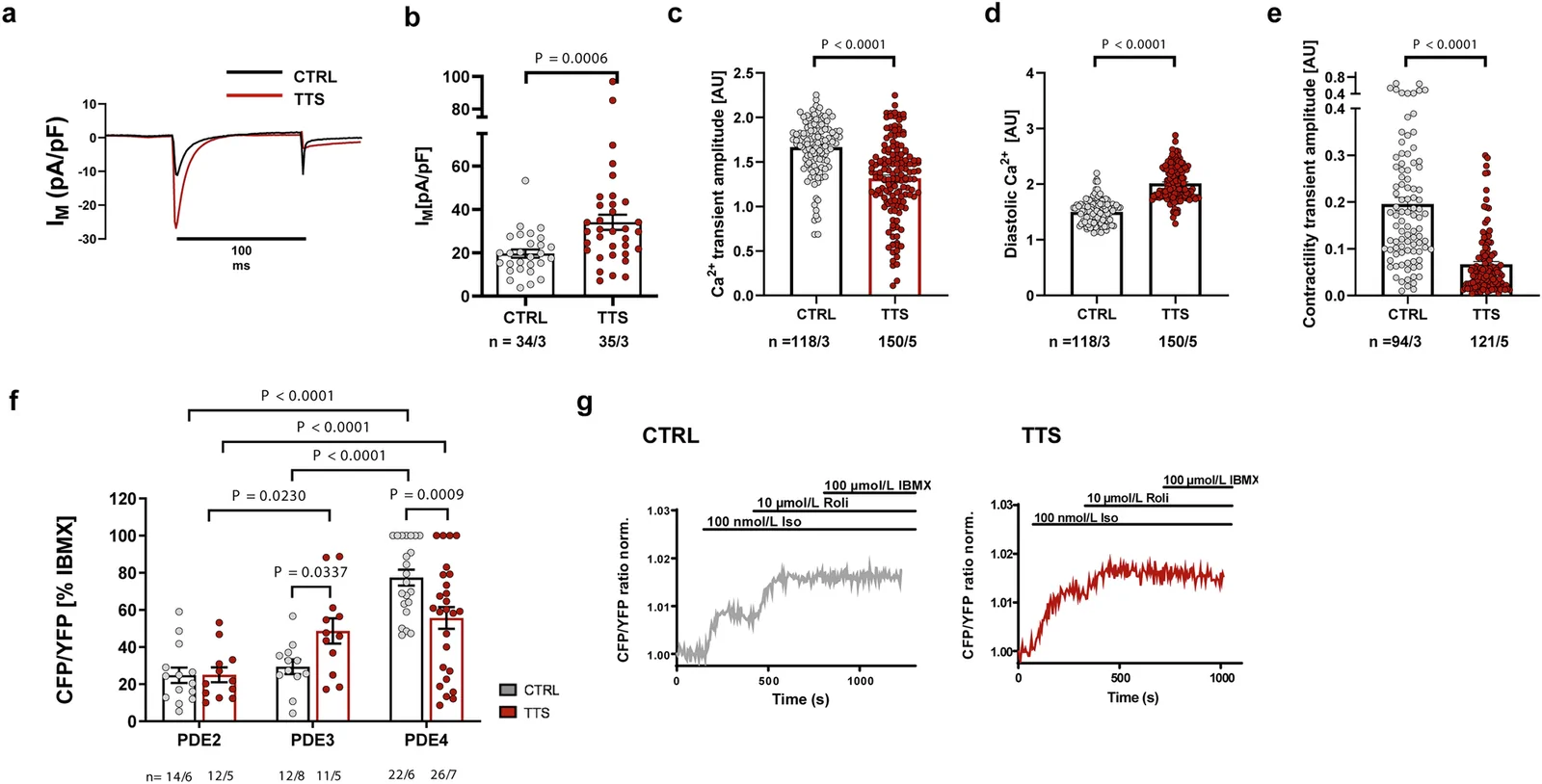
Ca^2+^ handling and PDE contribution in ventricular CTRL and TTS iPSC-CMs. **a**, **b** L-type Ca²⁺ currents in TTS iPSC-CMs. **a** Representative membrane current (I_M_) trace of L-type Ca^2+^ current (I_Ca,L_) in iPSC-CMs of CTRL and TTS. **b** Peak I_Ca,L_ (CTRL: *n* = 34 cells/3 diffs, TTS: *n* = 35/3) (Mann-Whitney’s test). **c**–**e** Ca^2+^ (Indo 1-AM epifluorescence microscopy) and contractility measurements (brightfield pixel correlation) on CTRL and TTS iPSC-CMs. **c**, **d** Ca^2+^ transient amplitude and diastolic Ca^2+^ at 0.75 Hz stimulation compared with CTRL. For control Ca^2+^ transient amplitude: baseline (*n* = 118/3); TTS Ca^2+^ transient amplitude: *n* = 150/5; CTRL diastolic Ca^2+^: *n* = 118/3; TTS diastolic Ca^2+^n = 150/5. (Mann-Whitney's test). **e** Contractility transient amplitude at 0.75 Hz stimulation in CTRL and TTS iPSC-CMs: CTRL: *n* = 94/3; TTS: *n* = 121/5. (Mann-Whitney’s test). **f** PDE2, PDE3, and PDE4 activity (as measured using an EPAC1-based FRET sensor for cAMP) after Iso treatment (100 nM) in TTS cells (PDE2: *n* = 12 /5, PDE3: *n* = 11/5, PDE4: *n* = 26/7) and CTRL cells (PDE2: *n* = 14/6, PDE3: *n* = 12/8, PDE4: *n* = 22/6) (Two-way ANOVA with Post hoc Tukey test). Shown are cAMP responses to individual PDE inhibitors as a % of maximal response evoked by 100 µM of the pan-PDE inhibitor IBMX. **g** Representative CFP/YFP traces for ventricular CTRL- and TTS-iPSC-CMs. All Data are presented as mean values ± SEM

We previously revealed that AF can cause distinct functional and molecular remodelling of the human LV.^44^ To further investigate ventricular alterations in TTS patients and to compare to atrial CMs Ca^2+^ characteristics, ventricular iPSC-vCMs were generated from TTS patient 5 (Supplementary Fig. 1c, f).^18^ We performed L-type Ca^2+^ current (*I*_Ca,L_) measurements via patch-clamp recordings in CaV1.2-expressing iPSC-vCMs of TTS and CTRL and demonstrated an increase in *I*_Ca,L_ density in TTS iPSC-vCMs compared with healthy controls (Fig. 4a, b). Furthermore, Indo 1-AM epifluorescence microscopy demonstrated reduced Ca²⁺ transient amplitudes (Fig. 4c) and elevated diastolic Ca²⁺ levels (Fig. 4d) in TTS iPSC-vCMs, reminiscent of an HFrEF-like phenotype. These findings are in line with the reduced contractility observed in TTS iPSC-vCMs (Fig. 4e) but differ from the atrial Ca^2+^ handling phenotype (Fig. 2). Despite these differences in cytosolic Ca^2+^ handling in ventricular compared to atrial TTS iPSC-CMs, ventricular TTS iPSC-vCMs also exhibited reduced PDE4 activity after Iso treatment (Fig. 4f, g), comparable to the alterations in atrial iPSC-CMs (Fig. 3a, b).

### PDE4 activator MR-L2 and β-blocker metoprolol as therapeutic approaches in the treatment of TTS

**Fig. 5:**
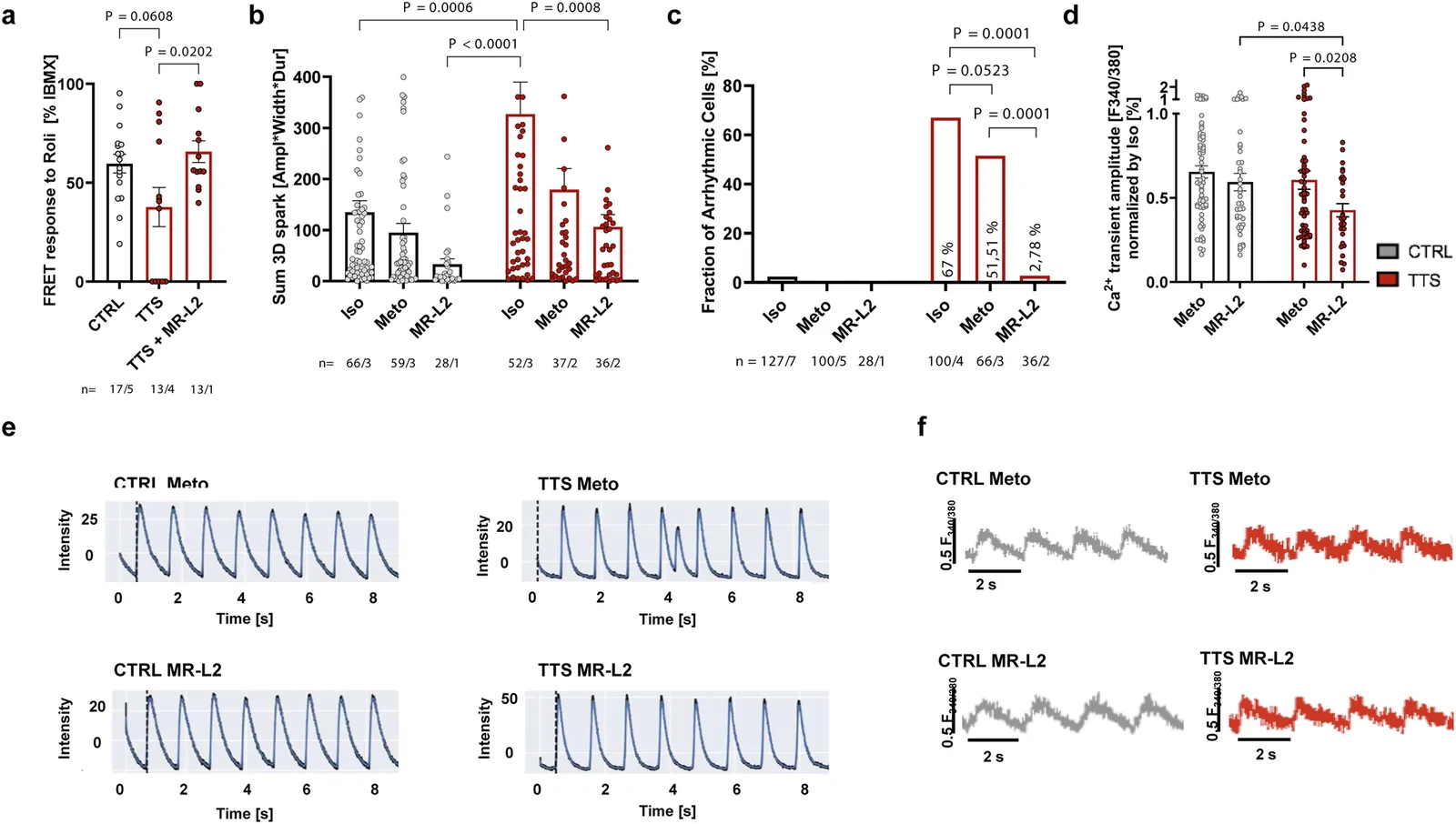
Treatment of atrial TTS iPSC-CMs with PDE4 activator MR-L2 and β-blocker metoprolol. **a** IPSC-CMs were stimulated with Iso (100 nM) followed by Roli and IBMX as described in Fig. 3a. Roli-sensitive (PDE4) activity is quantified relative to total PDE activity determined by IBMX. PDE4 activity in EPAC1-based FRET measurements after MR-L2 treatment (3 µM) of Iso-stressed TTS cells (*n* = 13 cells/1 diff) in comparison to CTRL cells without MR-L2 treatment (*n* = 17/5) and Iso stressed TTS iPSC-CMs without drug intervention (*n* = 13/4) (One-way ANOVA with Post hoc Tukey's test). **b, c** Diastolic Ca^2+^ leakage (confocal microscopy, Fluo 4-AM) (**b**) and amount of arrhythmic cells (transients and analysis of arrhythmic cells based on confocal microscopy line scans (Fluo 4-AM)) (**c**) in TTS iPSC-CMs (Ca^2+^ sparks: Iso: *n* = 52 cells/3 diffs; Iso + metoprolol: *n* = 37/2; Iso + MR-L2: *n* = 36/2; Fraction of arrhythmic cells: Iso: *n* = 100/4; Iso + metoprolol: *n* = 66/3; Iso + MR-L2: *n* = 36/2) and CTRL iPSC-CMs (Ca^2+^ sparks: Iso: *n* = 66/3; Iso + metoprolol: 59/3; Iso + MR-L2: 28/1; Fraction of arrhythmic cells: Iso: 127/7; Iso + metoprolol: 100/5; Iso + MR-L2: 28/1), **b** Two Way ANOVA with Post hoc Tukey test, **c** Fisher’s exact test analysis used for comparison of two groups (TTS Iso vs Meto, TTS Iso vs MR-L2; TTS Meto vs MR-L2). **d** Ca^2+^ transient amplitude normalised by Iso in TTS iPSC-CMs after metoprolol treatment (*n* = 61 cells/ 3 diffs) in comparison to MR-L2 treatment (*n* = 29/3). (CTRL iPSC-CMs: metoprolol (*n* = 66/4), MR-L2 treatment (*n* = 42/4) (Two-way ANOVA with Post hoc Tukey's test). **e** Exemplary Ca^2+^ transients in arrhythmia analysis of the CTRL and the TTS cell line after metoprolol and MR-L2 treatment. **f** Representative line scans of epifluorescence Ca^2+^ measurements of the CTRL and the TTS cell line after metoprolol and MR-L2 treatment. **b**–**f** Cells were paced at 1 Hz. All Data are presented as mean values ± SEM

Since PDE4 plays a crucial role in regulating cAMP signalling and its activity is downregulated in TTS patients in our study (Fig. 3), we analysed the effects of PDE4 activation and the resulting functional consequences in TTS. MR-L2 is an activator of long-form PDE4 isoforms, mimicking the effect of PKA phosphorylation and thereby decreasing intracellular cAMP levels.^28^ We compared these effects to those of metoprolol, a β_1_-AR-selective β-blocker commonly used in patients with TTS. MR-L2 increased PDE4 activity in Iso-treated TTS cells to levels observed in CTRL cells (Fig. 5a). Furthermore, PDE4 activation reduced the increased diastolic SR Ca^2+^ leak by 3-fold, whereas metoprolol reduced it by only 1.8-fold (Fig. 5b). Interestingly, while metoprolol had only a minor effect on arrhythmias in Iso-stressed TTS cells, these were largely abolished by MR-L2 (from 67% to 3% arrhythmic cells; Fig. 5c). Lastly, MR-L2 led to a greater reduction in the Iso-induced increase of the cytosolic Ca^2+^ transient amplitude compared to metoprolol (Fig. 5d). Ca^2+^ transients in arrhythmia analysis and representative line scans in epifluorescence measurements after treatment of the cells with metoprolol and MR-L2 are illustrated in Fig. 5e, f.

### Genetic variants in AHNAK in TTS patients

Since we previously identified genetic variants in TTS patients,^18^ we hypothesised that genetic factors may contribute to susceptibility to TTS. We included two additional TTS patients from our TTS stem cell cohort and performed whole-exome sequencing (WES) and Sanger sequencing on DNA extracted from primary skin fibroblasts of 6 TTS patients in total. We focused our WES data analysis on 81 genes previously associated with genetic forms of cardiomyopathies and cardiac arrhythmia because of the limited number of TTS patients. Using specific filter criteria, we found deleterious genetic variants in *AHNAK1* or *AHNAK2* in 83% of our TTS stem cell cohort (in 5 out of 6 patients) (Table 2). TTS patients 1, 2, 5, and 9 harbour genetic variants in different domains of *AHNAK1* and *AHNAK2*, which are predicted to replace highly conserved amino acids. These variant frequencies are present in around 0,001 in the gnomAD database, excluding those that represent single-nucleotide polymorphisms common in the general population (Table 2). Identifying variants in *AHNAK*, which encode proteins involved in Ca²⁺ and β-adrenergic signalling, in nearly all TTS patients of this study suggests a functional and cellular predisposition by these variants.

**Table 2:**
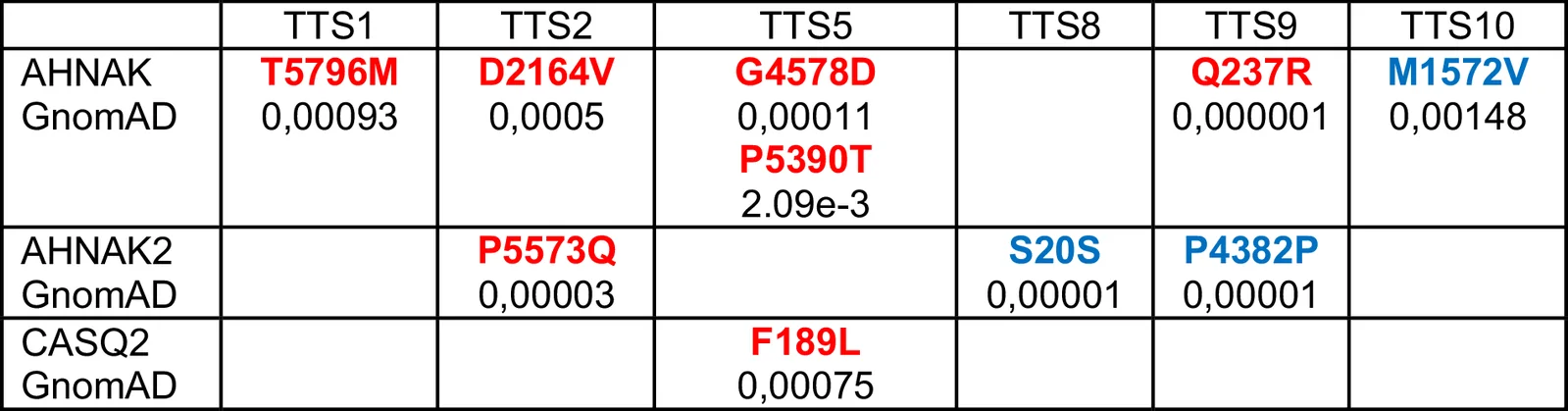
Overview of *AHNAK, AHNAK2* and *CASQ2* mutations identified in different TTS patients (red: deleterious, blue: neutral) and the frequencies in gnomAD-v4.1.1

The TTS patient 5 carries the heterozygous rare variant c.567 C > G (p.F189L) in the *CASQ2* gene (present in 0,00075 in the gnomAD database)^18^ in addition to two *AHNAK1* variants predicted to be deleterious or damaging. The heterozygous *AHNAK* variant p.P5390T is present in 3375 of 1614132 alleles (allele frequency 2.09e-3), and the number of homozygotes in the gnomAD database is 38. Since TTS is a multifactorial disease and this variant is localised at the C-terminal end of AHNAK, which is responsible for the interaction with the Ca^2+^ channel Cav1.2,^33,47^ we hypothesised that the homozygous *AHNAK* variant p.P5390T may impair AHNAK-Cav1.2 interaction, leading to enhanced Ca^2+^ influx as shown in Fig. 4a and, consequently, a higher arrhythmogenic potential in TTS. Therefore, in this work, we focused on the variants *AHNAK1* (p.P5390T, homozygous) and *CASQ2* (p.F189L, heterozygous) and generated three new cell lines utilising CRISPR/Cas9 genome editing. We introduced the heterozygous p.F189L *CASQ2* (c.567 C > G) mutation into a healthy CTRL cell line^18^ (CTRL-CASQ2), as published recently.^31^ Furthermore, we introduced the *AHNAK1* mutation (p.P5390T; c.16168 C > A) homozygously into the CTRL cell line (CTRL-AHNAK) and performed a genetic rescue of the same *AHNAK* mutation in the TTS cell line (TTS-res-AHNAK). Successfully gene-edited clones were confirmed by Sanger sequencing (Supplementary Fig. 3a). Those clones showed the integration of the *AHNAK* mutation from C-C-T (Pro) to A-C-T (Thr) at position p.5390 (CTRL-AHNAK), the *CASQ2* mutation from T-T-C (Phe) to T-T-G (Leu) at position p.189,^31^ and the rescue of the *AHNAK* mutation from C/A-C-T (Pro/Thr) to C-C-T (Pro) at position p.5390 (Supplementary Fig. 3a).

The CRISPR-edited cell lines CTRL-AHNAK and TTS-res-AHNAK, which have not been previously described, were characterised for their pluripotency and genetic integrity. The spontaneous differentiation potential of all generated iPSC lines was evaluated through in vitro embryoid body formation. Immunofluorescence analysis confirmed the expression of proteins representing all three germ layers: α-smooth muscle actin (α-SMA) for mesoderm, α-fetoprotein (AFP) for endoderm, and β-III-tubulin for ectoderm (Supplementary Fig. 3b). All iPSC lines exhibited characteristic pluripotent morphology, tested positive for alkaline phosphatase (ALP) staining, and expressed common pluripotency markers, including OCT4, SOX2, LIN28, TRA1-60 and NANOG, at the protein level (Supplementary Fig. 3c). To assess genomic integrity, standard G-banding karyotype analysis was conducted, revealing a normal karyotype (Supplementary Fig. 4). The five most probable hits in the off-target analysis of each genome-edited cell line were calculated by the Jefferson “Off-spotter” (Supplementary Fig. 5).

In conclusion, the newly generated CRISPR lines demonstrated pluripotency characteristics without genetic aberrations. These three genome-edited cell lines (CTRL-CASQ2, CTRL-AHNAK, and TTS-res-AHNAK) were differentiated into atrial iPSC-CMs. The expression of *CASQ2* and *AHNAK1* was confirmed at the mRNA level in atrial iPSC-CMs, with no significant differences in the expression of *CASQ2* in all cell lines but decreased expression of *AHNAK1* in atrial TTS iPSC-CMs in comparison to CTRL iPSC-CMs and TTS-res-AHNAK iPSC-CMs (Supplementary Fig. 6).

### The impact of the genetic variants in the *AHNAK* and *CASQ2* genes on arrhythmogenesis in TTS

**Fig. 6:**
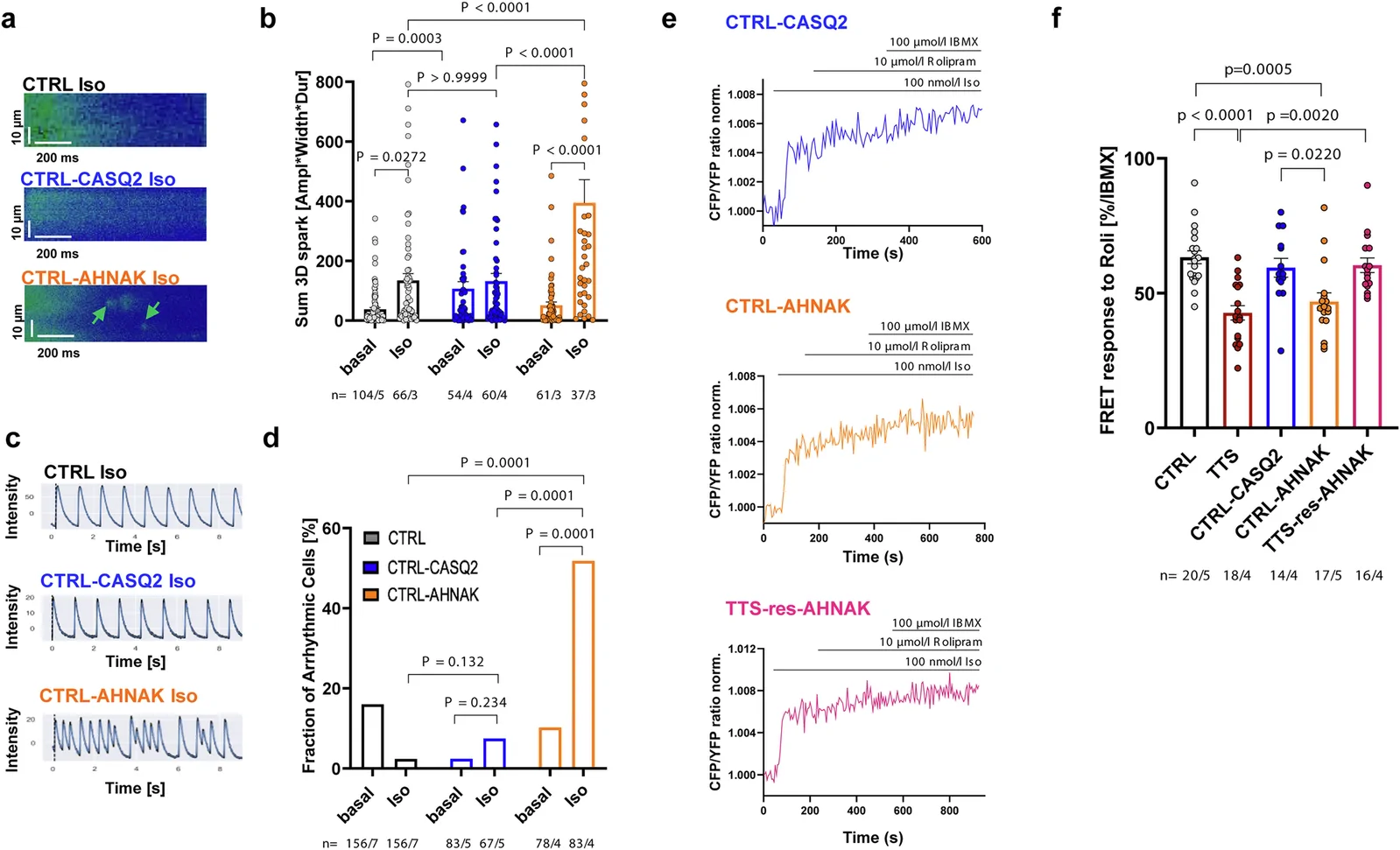
Diastolic SR Ca^2+^ leak, arrhythmias and PDE4 activity in genome-edited cell lines. **a** Exemplary line scans of a CTRL, a CTRL-CASQ2 and a CTRL-AHNAK cell after Iso treatment (100 nM) (green: cytosolic Ca^2+^, blue: background, arrows: Ca^2+^ sparks; scale bar: height = 10 µm, length = 200 ms). **b** Amount of Ca^2+^ sparks in CTRL, CTRL-CASQ2 and CTRL-AHNAK cells at baseline level (CTRL: *n* = 104/5; CTRL-CASQ2: *n* = 54/4; CTRL-AHNAK: *n* = 61/3) and after Iso (100 nM) treatment (CTRL: *n* = 66/3; CTRL-CASQ2: *n* = 60/4; CTRL-AHNAK: *n* = 37/3) (Two-way ANOVA with Post hoc Tukey test). **c** Exemplary line scan arrhythmia analysis of a CTRL, a CTRL-CASQ2 and a CTRL-AHNAK cell after Iso treatment. **d** Number of arrhythmic cells in CTRL, CTRL-CASQ2 and CTRL-AHNAK cells on baseline level (CTRL: *n* = 156/7; CTRL-CASQ2: *n* = 83/5; CTRL-AHNAK: *n* = 78/4) and after Iso (100 nM) treatment (CTRL: *n* = 156/7; CTRL-CASQ2: *n* = 67/5; CTRL-AHNAK: *n* = 83/4), Fisher’s exact test analysis used for comparison of two groups (CTRL Iso vs CTRL-CASQ2 Iso; CTRL Iso vs CTRL-AHNAK Iso; CTRL-CASQ2 Iso vs CRTL-AHNAK Iso; CTRL AHNAK basal vs CTRL AHNAK Iso; CTRL CASQ2 Iso vs CTRL AHNAK Iso; CTRL CASQ2 basal vs CTRL CASQ2 Iso). **e** Representative transients of the cAMP-fluorescence intensity in Iso-treated CTRL-CASQ2, CTRL-AHNAK and TTS-res-AHNAK cells following PDE4 inhibition, before treatment with the pan-PDE inhibitor IBMX. **f** The Rolipram (Roli)-sensitive fraction (PDE4) is presented as a percentage of total PDE activity, as assessed by the IBMX response (CTRL iPSC-CMs (*n* = 20 cells/5 diffs), TTS iPSC-CMs (*n* = 18/4), CTRL-CASQ2 iPSC-CMs (*n* = 14/4), CTRL-AHNAK cells (*n* = 17/5), TTS-res-AHNAK cells (n = 16/4). Kruskal-Wallis test with Dunn’s multiple comparisons. **a**, **b** Confocal microscopy (Fluo 4-AM); **c**, **d** Transients and analysis of arrhythmic cells based on confocal microscopy line scans (Fluo 4-AM); **a**–**d** Cells paced at 1 Hz. **e**, **f** Inverted fluorescence microscopy (EPAC1-based FRET sensor for cAMP). All Data are presented as mean values ± SEM

To better understand why TTS patients develop arrhythmias and exhibit decreased PDE4 activity under catecholamine stress, we differentiated the genome-edited cell lines into atrial iPSC-CMs. Consistent with the phenotype observed in TTS cells (Fig. 1a, b), atrial CTRL-AHNAK iPSC-CMs showed higher diastolic SR Ca^2+^ leak upon Iso treatment than CTRL and CTRL-CASQ2 cell lines (Fig. 6a, b). Accordingly, 52% of the Iso-treated CTRL-AHNAK cells exhibited arrhythmic beating, compared to less than 10% in CTRL and CTRL-CASQ2 cell lines (Fig. 6c, d).

Genome-edited cell lines were analysed for PDE4 activity using FRET measurements. The increase in cytosolic cAMP induced by PDE4 inhibition was suppressed in CTRL-AHNAK compared to CTRL and CTRL-CASQ2, similar to TTS cells (Fig. 6e, f). Of note, this reduced PDE4 activity could be restored in TTS cells with the *AHNAK* mutation rescued (Fig. 6f), supporting that the genetic variant p.P5390T in *AHNAK* reduced PDE4 activity under catecholamine stress. Under baseline conditions, no significant differences in PDE4 activity could be observed in any of the measured genome-edited cell lines (Supplementary Fig. 2b). Together, these data suggest that AHNAK-P5390T in TTS reduces PDE4 activity, increases diastolic SR Ca2+ leak, and increases susceptibility to arrhythmias in response to β-adrenergic stimulation.

**Fig. 7:**
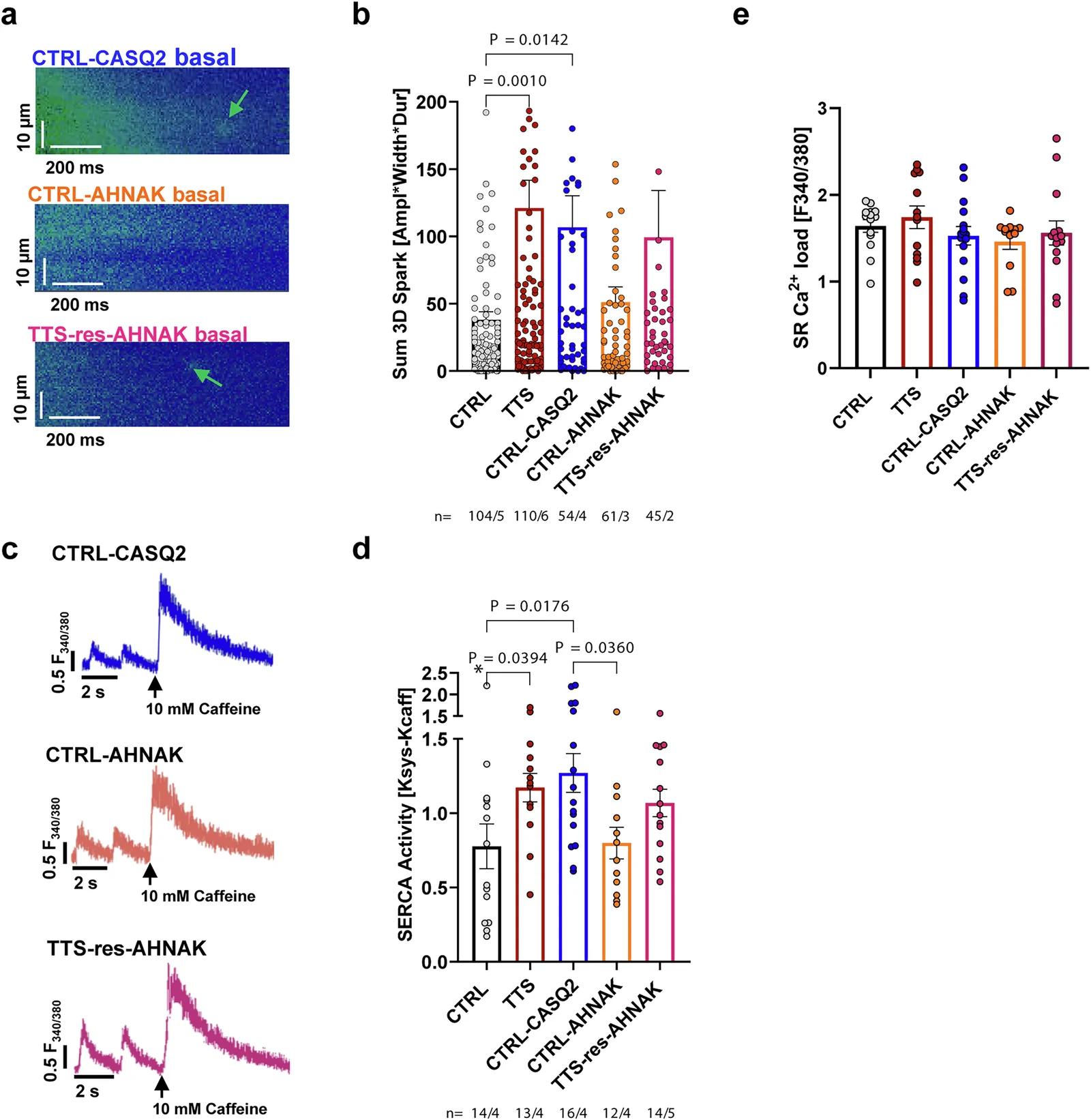
Diastolic SR Ca^2+^ leak, SERCA activity and SR Ca^2+^ load under baseline conditions. **a** Exemplary line scans of a CTRL-CASQ2, a CTRL-AHNAK, and a TTS-res-AHNAK cell under baseline conditions (green: cytosolic Ca^2+^, blue: background, arrows: Ca^2+^ sparks; scale bar: height = 10 µm, length = 200 ms). **b** Amount of Ca^2+^ sparks under baseline conditions in CTRL iPSC-CMs (*n* = 104/5), TTS iPSC-CMs (*n* = 110/6), CTRL-CASQ2 iPSC-CMs (*n* = 54/4), CTRL-AHNAK iPSC-CMs (*n* = 61/3) and TTS-res-AHNAK iPSC-CMs (*n* = 45/2). (Kruskal-Wallis test with Dunn correction). **c** Exemplary Caffeine (10 mM) transients of a CTRL-CASQ2 (blue), a CTRL-AHNAK (orange), and a TTS-res-AHNAK cell (magenta) (scale bar: height = 0.5F_340/380_, length = 2 s). **d** SERCA2a activity (K_sys_-K_caff_) in CTRL iPSC-CMs (*n* = 14/4), TTS iPSC-CMs (*n* = 13/4), CTRL-CASQ2 iPSC-CMs (*n* = 16/4), CTRL-AHNAK iPSC-CMs (*n* = 12/4) and TTS-res-AHNAK iPSC-CMs (*n* = 14/5). To assess overall group differences, group comparisons were performed using One-Way ANOVA (Sidak multiple comparisons). Additionally, exploratory pairwise comparisons using unpaired t-tests were performed to further investigate specific group differences. Significant differences are marked with *. **e** SR Ca^2+^ load was measured after Caffeine (10 mM) application (CTRL (*n* = 14/4), TTS (*n* = 13/4), CTRL-CASQ2 (*n* = 16/4), CTRL-AHNAK (*n* = 12/4), TTS-res-AHNAK (*n* = 14/5)). **a**, **b** Confocal microscopy (Fluo 4-AM), cells paced at 1 Hz; **c**–**e** Epifluorescence microscopy (Fura 2-AM), cells paced at 0.5 Hz. All Data are presented as mean values ± SEM

Under baseline conditions, no difference in the amount of Ca^2+^ sparks and the fraction of arrhythmic cells could be observed between atrial CTRL-AHNAK iPSC-CMs and CTRL cells. In contrast, CTRL-CASQ2 iPSC-CMs harbouring the *CASQ2* mutation F189L presented with a 2.8-fold higher diastolic SR Ca^2+^ leak than the CTRL under baseline conditions, as observed before in TTS iPSC-CMs (Fig. 7a, b). The SERCA2a activity was calculated from caffeine transients (Fig. 7c), showing a 1.6-fold increase in CTRL-CASQ2 cells in comparison to CTRL cells (Fig. 7d), whereas no significant differences in SR Ca²⁺ content were observed between the cell lines (Fig. 7e). These data suggest that CASQ2-F189L may contribute to increased diastolic SR Ca^2+^ leak and heightened SERCA2a activity in TTS cells under baseline conditions.

## Discussion

Atrial fibrillation (AF) occurs in up to one quarter of TTS cases^4–6^ and is associated with increased mortality,^4^ thromboembolic events and HF exacerbation.^6,48^ Previous studies revealed an association of QTc prolongation with arrhythmias and arrhythmic complications in TTS,^49^ but the pathophysiology of AF in TTS remains unclear. As accumulating evidence points to a genetic predisposition to TTS,^18,50^ we used atrial stem cell models from TTS patients and revealed that the AHNAK p.P5390T variant impairs β-AR-dependent PDE4 activation, thereby potentiating cAMP formation. Excessive cAMP-PKA signalling enhances SERCA activity, SR Ca^2+^ load and RyR2-mediated spontaneous Ca^2+^ release, all well-established triggers for arrhythmias. Therapeutically, PDE4 activation had greater antiarrhythmic efficacy than the β-blocker metoprolol, the current standard treatment for atrial arrhythmias in TTS.

Arrhythmias require both a *trigger* and a *substrate*. The *substrate* often arises from atrial enlargement, fibrosis, and electrical remodelling, slowing conduction that promotes electrical re-entry,^51^ while *triggers* arise from spontaneous CM action potentials (APs) caused by RyR2-mediated SR Ca²⁺ release and delayed afterdepolarisations (DADs).^24^ CMs from patients with chronic or paroxysmal AF exhibit spontaneous APs mainly due to DADs triggered by spontaneous RyR2-mediated SR Ca²⁺ release (Ca²⁺ sparks).^52,53^ While SR Ca^2+^ load is unchanged in chronic AF, it is increased in paroxysmal AF, related to SERCA2 hyperactivation from PKA-mediated phospholamban hyperphosphorylation.^52,53^ In the present study, TTS iPSC-CMs had a phenotype similar to adult CMs from patients with paroxysmal AF: PKA-mediated phospholamban hyperphosphorylation, increased SERCA2 activity, and accelerated diastolic Ca^2+^ decay. This, likely together with RyR2 dysfunction, resulted in increased Ca^2+^ sparks, predisposing to β-AR-induced arrhythmias, despite reduced diastolic Ca^2+^ concentrations. This is in line with previous mouse studies which revealed that SERCA2 overexpression increases SR Ca^2+^ load and exacerbates RyR2-mediated arrhythmias.^32^

However, Ca^2+^ handling alterations in *atrial* TTS iPSC-CMs differed from *ventricular* TTS iPSC-CMs, which showed *reduced* cytosolic Ca^2+^ transient amplitude and contractility despite increased I_Ca,L_-mediated Ca^2+^-influx, suggesting disturbed coupling between L-type Ca^2+^ channels and RYR2 and/or decreased SR Ca^2+^ load. Furthermore, diastolic Ca^2+^ was *increased* in ventricular TTS iPSC-CMs, likely reflecting increased SR Ca^2+^ leak in TTS. The ventricular alterations are reminiscent of alterations in ventricular myocytes from patients with HFrEF,^54^ but also of myocytes after irregular pacing, as it occurs in AF.^44^ Since irregular pacing aggravates defects in Ca^2+^ handling in HFrEF,^55^ AF may similarly deteriorate Ca^2+^ handling in ventricular TTS myocytes and thereby LV function, the typical hallmark of TTS.

In addition, TTS-iPSC‑CMs exhibited APD prolongation, predisposing to early afterdepolarisations (EADs).^51^ Together, these changes suggest that both altered repolarisation and disturbed Ca^2+^ cycling contribute to the arrhythmia-prone phenotype in TTS, with EAD- and DAD-associated mechanisms likely operating in parallel. Although we observed shortened field potential duration in TTS at baseline, likely related to higher spontaneous beating frequency, these differences disappeared with β-adrenergic stimulation. Therefore, the proarrhythmic phenotype is likely driven primarily by impaired Ca^2+^ handling and prolonged APD rather than altered electrical propagation.

The increased PKA-mediated phosphorylation of PLN Ser16 in TTS suggests a pre-activated adrenergic state in TTS cells and supports the concept that aberrant catecholamine signalling contributes to arrhythmogenicity in atrial TTS CMs.^51,56^ Although contractile function is governed primarily by PDE3 in human CMs, PDE4 further fine-tunes microdomain cAMP levels, thereby limiting excessive SR Ca²⁺ release.^25^ In our studies, both atrial and ventricular TTS-iPSC‑CMs exhibited decreased PDE4 activity after Iso treatment. This is in line with previous studies on human CM from patients with AF, in which reduced PDE4 activity contributes to arrhythmias.^22^ Furthermore, studies in murine cardiac myocytes revealed that deficiencies in both PDE4B and PDE4D heighten ventricular arrhythmia risk,^57^ with PDE4B localised near caveolin-rich membranes and RyR2, and PDE4D regulating the SERCA2a microdomain.^58^ Together, these results indicate that reduced β-AR-induced PDE4 activity in atrial TTS CMs can cause cellular arrhythmias as a trigger for AF.

In this study, we found genetic variants in *AHNAK* in 83% of analysed TTS patients (Table 2). AHNAK is a 700 kDa scaffold protein involved in blood-brain barrier formation,^59^ cell structure, migration^60,61^ and cardiac Ca^2+^ channel regulation via Cav1.2 inhibition.^33^ β-adrenergic stimulation increases Ca²⁺ currents by phosphorylating AHNAK, an effect also observed with *AHNAK* variants such as p.I5236T.^62^ We identified the AHNAK amino acid substitution p.P5390T in TTS patient 5 in the C-terminal region of the protein, responsible for binding Cav1.2.^33^ We used genome-edited isogenic *AHNAK* variant insertion iPSC-CMs and demonstrated that AHNAK-P5390T reduces PDE4 activity, increases diastolic SR Ca^2+^ leak, and increases susceptibility to arrhythmias in response to β-adrenergic stimulation, comparable to TTS iPSC-CMs. A rescue of AHNAK-P5390T normalised PDE4 activity to CTRL levels, supporting a link between the *AHNAK* variant and reduced PDE4 activity in TTS cells. To the best of our knowledge, no direct interaction between AHNAK and PDE4 isoforms has been reported to date. However, PDE4 isoforms localise to subcellular microdomains whose disruption promotes arrhythmias.^63^ As a scaffolding protein, AHNAK may help maintain these domains,^61,64^ although a direct AHNAK-PDE4 interaction remains to be established in the future. AHNAK regulates Cav1.2 β₂-subunit availability and thereby I_Ca,L_-dependent Ca²⁺ influx and SR Ca²⁺ release.^33^ Its impairment may disrupt Ca²⁺ homoeostasis in TTS, particularly during β-adrenergic stimulation, thereby promoting arrhythmias. TTS patient 5 harbours, in addition, the variant F189L in *CASQ2* responsible for heightened diastolic Ca^2+^ leakage under baseline conditions. This is in line with previous studies on Casq2⁻/⁻ CPVT mice, which show increased spontaneous SR Ca²⁺ release.^30^ The highly conserved *CASQ2* F189L variant affects cytosolic Ca^2+^ dynamics and hERG channel function, supporting its functional relevance in TTS. In conclusion, together with previously reported hERG variants in TTS,^16^ our findings indicate that AHNAK p.P5390T and CASQ2 p.F189L perturb PDE4-dependent cAMP signalling and Ca²⁺/ion-channel homoeostasis, respectively, thereby increasing arrhythmogenic susceptibility both at baseline and during β-adrenergic stimulation. Importantly, the identified variants should not be interpreted as causal drivers of TTS but rather as modifiers of cellular responses to catecholaminergic stress, contributing to an arrhythmogenic substrate. Thus, our data support a mechanistically plausible but not causal relationship among genetic variants, altered PDE/cAMP regulation, and arrhythmic events in TTS CMs.

No randomised prospective trials exist for TTS treatment. β-blockers are frequently administered, despite limited evidence.^11^ Recent data suggest β-blockers reduce long-term mortality, but not TTS recurrence.^13^ Metoprolol partially improved Ca^2+^ homoeostasis in catecholamine-stressed atrial TTS iPSC-CMs by reducing diastolic Ca^2+^ leak, arrhythmia susceptibility, and abnormal Ca^2+^ transients. However, its therapeutic potential in TTS requires further investigation. Studies have investigated PDE upregulation as a therapeutic strategy in cardiac disease. Pavlaki et al. showed that upregulation of PDE4B3 via gene therapy restored compartmentation in caveolin-rich plasma membrane and RYR microdomains.^65^ Also, Molina et al.'s study suggests new compounds targeting PDE4 activity could potentially prevent atrial contractile dysfunction and AF development.^22^ A novel PDE4 activator, recently explored for polycystic kidney disease, shows promise in restoring cAMP balance in diseases characterised by increased cAMP levels.^28^ In this study, we tested the PDE4 activator MR-L2, as described by Omar et al.,^28^ on atrial TTS iPSC-CMs and found higher efficacy in normalising altered Ca^2+^ parameters compared to metoprolol treatment. MR-L2 lowered elevated diastolic Ca^2+^ leak 1.6 times as much as the β-blocker. These results highlight MR-L2 as a potential patient-specific therapy.

This study’s TTS patient had no clinical atrial or ventricular arrhythmias, but presented with marked QTc prolongation, suggesting an underlying pro-arrhythmogenic cellular phenotype as previously shown by our group. While QTc prolongation in TTS is primarily linked to ventricular arrhythmogenesis, the effects on atrial CMs and the role of subclinical atrial remodelling in atrial arrhythmogenesis require further investigation. iPSC-CMs do not fully attain adult-like cardiac maturity and retain immature features, including incomplete Ca²⁺ cycling and β-adrenergic signalling.^66^ Within this limitation, we observed altered electrophysiological properties, Ca^2+^ handling, and β-adrenergic responses in TTS iPSC-CMs. These findings likely reflect intrinsic, disease-related differences within the iPSC-CMs maturation spectrum rather than true adult-like maturation. Importantly, all cell lines were generated and analysed under identical conditions. A potential limitation is the lack of related healthy controls, which may raise concerns about genetic background and incomplete penetrance. We addressed this using isogenic rescue and knock-in lines, isolating mutation-specific effects. Future studies could explore familial lines to further dissect genetic modifiers of penetrance. Given the limited number of patients included here, this study represents a thorough patient-specific investigation and cannot draw conclusions about broader TTS populations or therapeutic relevance. Larger genetic studies are needed to determine whether *AHNAK* and *CASQ2* variants are enriched in TTS and contribute to disease susceptibility at the population level beyond the cellular mechanisms demonstrated here. Functional characterisation was restricted to the AHNAK p.P5390T variant, while other identified variants, including p.G4578D, were not investigated experimentally. The p.P5390T is relatively frequent in the general population (3.375 out of 1.614.132 alleles and even 38 homozygous carriers), allowing us to study hetero- or homozygosity of a common predisposing variant in the multifactorial disease TTS. Further, the predicted functional impact and localisation of p.P5390T within the C-terminal Cav1.2-interacting region of *AHNAK* made it the most compelling candidate for mechanistic analysis.

**Fig. 8:**
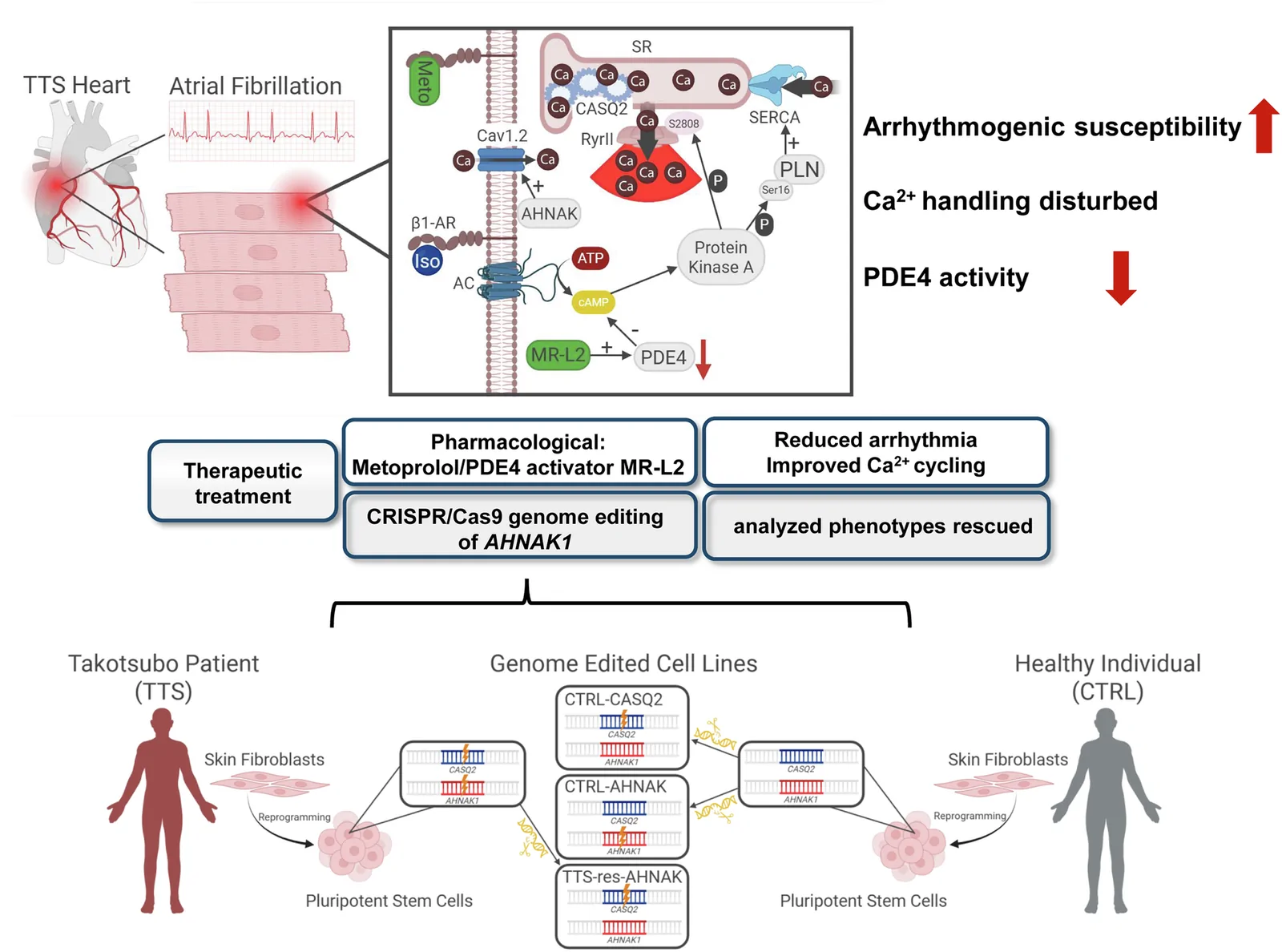
Schematic representation of the pathophysiological mechanisms in a Takotsubo (TTS) patient. The cellular and molecular mechanisms that may contribute to arrhythmogenesis and potential therapeutic strategies were investigated using atrial and ventricular linduced pluripotent stem cell-derived cardiomyocytes (iPSC-CMs) from this TTS patient and genome-edited iPSC lines (*AHNAK* and *CASQ2*). To induce stress and simulate a TTS event, isoprenaline (Iso) was applied to activate β1-adrenoceptors (ARs) and initiate cyclic adenosine monophosphate (cAMP)-mediated activation of protein kinase A (PKA), leading to the subsequent phosphorylation of key target proteins, such as phospholamban (PLN) and sarcoplasmic reticulum Ca^2+^ ATPase (SERCA). Iso-treatment resulted in heightened arrhythmias and altered Ca^2+^ handling in TTS  and CTRL-AHNAK iPSC-CMs. To mitigate these effects pharmacologically, the β-blocker metoprolol (meto) was applied, leading to a partial normalisation in Ca^2+^ parameters. Additionally, TTS cells exhibited decreased phosphodiesterase 4 (PDE4) activity. Therapeutic activation of PDE4 with MR-L2 improved the impaired parameters in TTS iPSC-CMs. Created in BioRender.com

Taken together, our data suggest that arrhythmogenic risk in TTS is driven by a combination of disturbed Ca²⁺ homoeostasis, reduced PDE4 activity, and genetic susceptibility. In particular, the AHNAK p.P5390T variant appears to contribute to catecholamine-induced cellular arrhythmic phenotypes, highlighting AHNAK as a potential modifier of TTS-related arrhythmogenesis. Drug intervention with the PDE4 activator MR-L2 restored PDE4 activity and improved arrhythmogenic behaviour and selected abnormal Ca²⁺ handling more effectively than the clinically used β-blocker metoprolol in atrial iPSC-CMs of TTS, indicating PDE4 modulation as a potential therapeutic avenue warranting further investigation (Fig. 8).

## Materials And Methods

### Institutional Review Board Statement

The study was conducted in accordance with the Declaration of Helsinki and approved by the local Ethics Committee of the University Medical Centre Göttingen (Az-10/9/15) and the Ethical Committee of the University of Zurich (KEK-No.: 2013-0075). Informed Consent Statement: Informed consent was obtained from all subjects involved in the study.

### TTS patients

The TTS patient in this study is registered in the International Takotsubo Registry (NCT01947621). This study used one healthy donor as a control subject (CTRL).^18^

### Reprogramming of somatic cells into iPSCs

Reprogramming of somatic cells into iPSCs was performed using Sendai Virus (for TTS)^67^ and a plasmid-based method (for CTRL),^68^ as described in previously published protocols.^18^ Briefly, skin fibroblast cultures were established from donor skin punch biopsies (3.5-4 mm) taken aseptically, placed in Dulbecco’s Modified Eagle Medium (DMEM) (Thermo Fisher Scientific) with penicillin (100 U/mL) and streptomycin (100 μg/mL). The biopsies were cut into 1-1.5 mm pieces, placed epidermis side up in a culture dish, and grown in fibroblast growth medium containing DMEM, 10% foetal calf serum (FCS), 1x non-essential amino acids (NEAA), glutamine (2 mM), β-Mercaptoethanol (50 μM), penicillin (50 U/mL), streptomycin (50 μg/mL), and basic fibroblast growth factor (bFGF) (10 ng/mL) at 37 °C with 5% CO_2_. Fibroblasts (3 × 10^5^) were seeded in 6-well or 12-well plates one day before transduction. The CytoTune™-iPS 2.0 Sendai Reprogramming Kit (Thermo Fisher Scientific) was used with a multiplicity of infection (MOI) of 5/5/3 (KOS/hc-myc/hKLF4) and added to the cells in fresh medium. The medium was changed every other day, and blood medium was supplemented with 500 μM sodium butyrate. On day 7, the cells were transferred to Geltrex-coated 6-well plates, and the medium was switched to Essential 8 (E8) medium. After 2-3 weeks, colonies with iPS-like morphology were manually picked to establish different stem cell lines. These new stem cell lines were passaged with Versene solution and cultivated in E8 medium.

For plasmid-based reprogramming, 5 × 10^5^ cells were electroporated using the NHDF Nucleofector Kit (Lonza) with 1 μg each of plasmids pCXLE-hSK, pCXLE-hUL, and pCXLE-hOct3/4-shp53-F. Electroporation was performed using the Nucleofector II (Lonza) with program P22 or U23. The cells were then plated in fibroblast medium with 5 μM pro-survival factor (Millipore) and 500 μM sodium butyrate. The medium was changed every other day. On day 7 post-transfection, fibroblasts were harvested and replated on Geltrex-coated 6-well plates to pick iPS-like colonies after 2-3 weeks.

The two cell lines used in this study were named TTS and CTRL.

### CRISPR/Cas9 genome editing

Previously, whole-exome sequencing (WES) was performed on DNA extracted from primary skin fibroblasts of the TTS patient, with data analysis focused on 81 genes known to be associated with genetic forms of cardiomyopathies and cardiac arrhythmias, revealing a mutation in the *CASQ2* gene.^18^ In addition to the identified mutation in the *CASQ2* gene in this TTS patient, a concurrent variant was also discovered in the *AHNAK*1 gene in the same patient. For further analysis of the impact of these mutations on arrhythmogenesis in TTS, we created three new cell lines utilising CRISPR/Cas9 genome editing.

To unravel the underlying mechanisms leading to TTS in a patient-specific approach, we introduced the heterozygous p.F189L Casq2 (c.567 C > G) mutation into our previously described healthy^18^ CTRL cell line (CTRL-CASQ2), as reported previously.^31^ Further, we introduced the *AHNAK1* mutation (p.P5390T; c.16168 C > A) into the CTRL cell line (CTRL-AHNAK, homozygously) and performed a genetic rescue of the same *AHNAK1* mutation in the TTS cell line (TTS-res-AHNAK). In total, 5 different iPSC lines were used in this study (CTRL, TTS, CTRL-CASQ2, CTRL-AHNAK, TTS-res-AHNAK) (Summary Fig. 8).

For generation of the iPSC line CRTL-AHNAK, the wildtype CTRL iPSC line was gene-edited with the crRNAfrom IDTdna: GCTTAGATCAGTAGCTCCTA. The used HDR template from IDTdna consisted of 91 base pairs: TGTCTCTGGTGACATCAAATGCCCTAAAGTATCCGTAGGAGCT-A-CTGATCTAAGCTTGGAGGCATCCGAAGGCAGCATTAAACTTCCCAAA. 2×10^6^ iPSCs were electroporated with 5 µl crRNA (100 µM), 5 µl tracrRNA (100 µM), 2 µl Cas9 protein (10 ng/µl), and 1 µl electroporation enhancer with the Human Stem Cell Nucleofector Kit (Amaxa VPH-5022) and the Amaxa Nucleofection II device (Lonza, program B-016). We cultured 32 clones until they reached the proper confluency, then snap-froze half of a 6-well plate for sequencing and the other half for cryopreservation. Through sequencing, we identified a clone with the homozygous insertion of the intended base exchange, which proved to include only the intended base exchange after multiple passages.

For generation of the iPSC line TTS-res-AHNAK, the TTS iPSC line was gene-edited with the crRNA from IDTdna: GATGCCTCCAAGCTTAGATC. The HDR template used from IDTdna consisted of 91 base pairs: GCTGTCTCTGGTGACATCAAATGCCCTAAAGTATCTGTAGGAGCT-C-CTGATCTAAGCTTGGAGGCATCCGAAGGCAGCATTAAACTTCCCA. Electroporation was performed in the same manner as described above.

We cultured 99 clones until they reached the proper confluency, then snap-froze half of a 6-well plate for sequencing and half for cryopreservation. Genomic DNA was isolated following the instructions of the QIAamp Mini Kit, and Sanger sequencing was performed at Microsynth Seqlab in Göttingen. We sequenced the five most probable hits calculated by the Jefferson “Off-spotter”. Primers for Sanger sequencing are given in Table 3.

**Table 3:** Primers used for Sanger sequencing

Gene name	Product size (bp)	Forward primer sequence	Reverse primer sequence	Annealing temperature	Cycle
*AHNAK1*	170	CCAGATGTGACACTGAGGGG	CCCGGAGTAGAGATGCCAAA	57 °C	35
*API5*	243	CACCGTGGCGTAACCTAACT	GAGTCCCTCTTCTGTTTCTCACA	56,4 °C	36
*BSN*	76	AGCTCTAGGACACAGAGG GAG	TCTAGGGCCTGCTGTCAAAG	56°C	36
*CASQ2*	463	CCGGTTCCCTCTGTTTCGT	AAAGAGGCAAGGGAGGATGGTT	56 °C	36
*C12orf42*	198	TTTGTACGTGCAGCTCTTCCT	TGTGCTTCTGGTAGTCTCAGC	56°C	36
*ECEC1*	950	ATGGTTTCCTGCTTCCACCC	AGCTCCCTCAGGGGTTCTAA	56°C	36
*FBXL7*	288	CTTGGTTCCCAGTAGGCACT	AGGAGTACATCAAGCCCATGC	55 °C	36
*JAZF1*	228	TGTTACCCTGGGACTGTGTG	GGAGGTGCATTCCAATCTGC	54 °C	36
*NKD1*	877	GCACCAGTCGAATGAGCGTT	GTTTGCCAAAGGACTCCCTG	54 °C	36
*TAF9*	262	AGACCCTCCAGGCAAGACAA	CATCTTACCCTTAGCCCCAGC	55 °C	36
*TUFT1*	173	ACCTCTAGAGCAAGGGAGAGA	TGTGTCCCGACTGTTCACCA	56°C	36
*TXLNG*	605	TCGCTGTCCTCCCAAGAATC	CCTGAACTCAAGGAGTCCCC	54 °C	36

### Cell culture

iPSCs were cultured at 37 °C and 5% CO_2_ in E8 medium (1x E8 medium, 1x E8 supplement (Thermo Fisher Scientific)) on 6-well plates (CytoOne). Once the cells reached 90% confluency, cell passaging was performed by incubating for 3–5 minutes with Versene (Thermo Fisher Scientific), followed by resuspension in E8 medium supplemented with 2 µM Thiazovivin (TZV) (Merck Millipore). Subsequently, the cells were transferred to newly coated plates.

As described in previously published papers, the Wnt signalling pathway was initially activated and then inhibited to promote differentiation into CMs.^69^ iPSCs were plated onto 12-well plates (CytoOne) at a density of 30,000 cells per well. Upon reaching 80-90% confluency, differentiation was induced using Cardio Differentiation medium (1x Roswell Park Memorial Institute (RPMI) 1640 medium + GlutaMAX (Thermo Fisher Scientific), 0,05% human recombinant Albumin (Sigma-Aldrich), 0,02% L-ascorbic Acid 2-Phosphate (Sigma-Aldrich)) supplemented with 4 µM CHIRR99021 (Merck Millipore) to inhibit Glycogen synthase kinase 3 (GSK3) (day 0). After 48 hours, the medium was replaced with Cardio Differentiation medium supplemented with 5 mM inhibitor of Wnt production-2 (IWP2) (Merck Millipore) to inhibit Wnt production and, in the case of atrial CMs, 1 µM retinoic acid (Sigma-Aldrich) was added on days 3 and 4 to ensure differentiation into atrial iPSC-CMs.^70^ Cell Culture was performed using Cardio Differentiation medium until the cells began to beat.

Cells were then plated onto new 6-well plates with an approximate density of 600,000 cells per well. For digestion, cells were incubated with 0.25% Trypsin-EDTA (Thermo Fisher Scientific) for five minutes, followed by the addition of inactivated FCS (Sigma-Aldrich) to stop the digestion. After centrifugation at 200 g for five minutes and resuspension in Cardio Digestion medium (80% Cardio Culture medium (1x RPMI 1640 Medium + GlutaMAX, 1x B27 Supplement (Thermo Fisher Scientific), 20% inactivated FCS, 2 µM TZV), the cells were plated onto 6-well plates.

According to protocols of previously published papers, the cells underwent a selection process after 48 hours, using Cardio Selection medium (1x RPMI 1640 medium (Thermo Fisher Scientific), 0,05% human recombinant Albumin, 0,02% L-ascorbic Acid 2-Phosphate, 4 mM Lactate/4-(2-hydroxyethyl)-1-piperazineethanesulfonic acid (HEPES) (Sigma-Aldrich)), which was changed every other day until pure cardiac morphology was observed.^71^ After the successful selection of iPSC-CMs, the cells were cultured in Cardio Culture medium for 60 days to ensure maturation before measurements.

To assess the differentiation into atrial iPSC-CMs, immunostainings of Myosin light chain 2 atrium (MLC2a) and Myosin light chain 2 ventricle (MLC2v) was performed, and the ratio of MLC2a- to MLC2v-stained cells was calculated by counting the number of cells positive for MLC2a and MLC2v, respectively. Only differentiations with more than 80% MLC2a-positive CMs were utilised for further atrial iPSC-CM experiments and 80% MLC2v-positive CMs for ventricular experiments. In minimum, 3 atrial/ventricular cardiac differentiations were used per analysis.

### Ca^2+^ Imaging of atrial iPSC-CMs

To evaluate diastolic SR Ca^2+^ sparks, 380,000 iPSC-CMs, plated on Geltrex-coated cover slips (R. Langenbrinck) one week before measurement, were first loaded with Fluo 4-Acetoxymethylester (Fluo 4-AM) (2.5 μM) (Invitrogen) supplemented with Pluronic F-127 0.05% w/v (Thermo Fisher Scientific) for 30 minutes, followed by a 15-minute incubation in Tyrode’s solution (5.4 mM KCl, 140 mM NaCl, 1 mM MgCl_2_, 10 mM HEPES, 10 mM glucose, 1.8 mM CaCl_2_, pH 7.4). An LSM 720 confocal microscope (Zeiss) equipped with a Myopacer device (IonOptix) was used for the measurement. The cells were paced at 1 Hz. The following settings using the software Zen 2009 were applied: argon ion laser excitation at 488 nm with 1.0 – 2.2%, a pinhole of 6 AU with 700 gain and offset 0. Using the line scan function, an area in the cytoplasm of a cell was selected and the line scan mode was set to 10.000 cycles, 12 bit unidirectional, zoom factor 3, 512 pixels, no delay and maximum scan speed (945.5 μs per line scan). The fluorescence images were recorded in the line-scan mode (512 pixels per line, scan width: 35.4 μm, pixel time: 0.64 μs, 10,000 unidirectional line scans/image, 6.9-second measurement period). Subsequently, diastolic Ca^2+^ sparks were analysed using the ImageJ SparkMaster program and the spark was calculated by summing the product of the amplitude, the width and the duration of each spark.

One week before measurements, 30,000 iPSC-CMs were plated onto Geltrex coated 35 mm Fluoro dishes (WPI). IPSC-CMs were incubated with Fura 2-Acetoxymethylester (5 µM, Fura 2-AM, Invitrogen) and 0.05% Pluronic F-127 0.05% w/v for 15 minutes and afterwards washed for 15 minutes with Tyrode’s solution (4 mM KCl, 140 mM NaCl, 1 mM MgCl_2_, 10 mM HEPES, 10 mM glucose, 1.25 mM CaCl_2_, pH 7.4). To calculate the Fura-2AM fluorescence ratio, we employed alternating excitation wavelengths at 340 nm and 380 nm and the emitted fluorescence was collected at 510 nm. All measurements were performed on an epifluorescence microscope (IonOptix) equipped with a Myopacer device, to allow for pacing of the iPSC-CMs during measurements. Fluorescence intensities were traced with the IonOptix software. The cells were stimulated at 30 V for 10 ms at 1 Hz. For Caffeine measurements, one cell was paced at 0.5 Hz and one drop (approximately 50 µl) of 10 mM Caffeine was added to assess sarcoplasmic Ca^2+^ content. Sarcoplasmic reticulum Ca^2+^-ATPase 2a (SERCA2a) activity was analysed by calculating the difference between the decay constants (K) of the systolic Ca^2+^ transient and the caffeine-induced Ca^2+^ transient (Ksys - Kcaff).^72^ The recorded Ca^2+^ transients were analysed using the IonWizard software (IonOptix Corp.).

Cells were measured under baseline conditions and other cells of the same dish after treatment of: Iso (10 nM, 100 nM, respectively, 5-minute incubation), metoprolol (5 µM, 10-minute incubation), Rolipram (10 µM, 10-minute incubation), MR-L2 (3 µM, 20 min incubation).

### Ca^2+^ and contractility measurements of ventricular iPSC-CMs

Functional analysis of TTS-iPSC-CMs and CTRL-iPSC-CMs was performed using Ca^2+^ imaging and contractility measurements with the IonOptix Calcium and Contractility System coupled to a Cytocypher inverted microscope (40x objective). Seven days before the measurements, 1–1.5 × 10^5^ iPSC-CMs were seeded onto Geltrex-coated 35 mm dishes (MatTek). Cells were incubated for 20 min at 37 °C in 1 ml Tyrode solution (140 mM NaCl, 5.4 mM KCl, 1 mM MgCl_2_, 10 mM HEPES, 10 mM glucose, 1.8 mM CaCl_2_) containing 1 µM Indo-1 AM (Thermo-Fischer) to stain free Ca^2+^. Cells that received only Tyrode served as a control condition. During the treatment time, 30 individual cells were selected prior to data acquisition.

After incubation with the treatment, cells were continuously perfused with Tyrode solution containing the respective treatment and electrically paced at increasing frequencies (0.5, 0.75 and 1 Hz). Simultaneous recordings of Ca^2+^ transients (Indo 1-AM epifluorescence microscopy) and contractility (brightfield pixel correlation) were performed for 17 sec at 0.5 Hz, 12.5 sec at 0.75 Hz, and 8.5 sec at 1 Hz per cell. Measurements were collected using IonWizard 6.3 and the resulting data were analysed with CytoSolver 2.2.2 and Microsoft Excel.

### Beating frequency and field potential duration

Multi-electrode array (MEA) recordings were used to assess beating frequency (BF) and field potential duration (FPD). Custom 6-well MEA plates were sterilised (70 °C water bath, 2 h; UV, 30 min), coated with 5 µl Geltrex, and incubated at 37 °C for 30 min. Cardiomyocytes (25,000 cells/well) were seeded onto the electrodes. After 7 days, recordings were performed 4 hour following medium change. Signals were acquired and analysed using MC Rack software; FPD was corrected using the Fridericia method.^73^ Baseline activity was recorded for 5 min, followed by sequential Iso (10 nM–1 µM) treatments with 5 min recordings each.

### Action Potential

Action potentials of human atrial iPSC-CMs were measured using a whole-cell patch clamp technique in current-clamp configuration (HEKA Electronic). Patch pipettes with tip resistances ranging from 2 to 4 MΩ were backfilled with internal solution containing (in mM): K-Aspartate 122, NaCl 10, KCl 8, MgCl2 1, Mg-ATP 5, HEPES 10 (pH 7.2, KOH). The iPS-CMs subjected to AP recordings were placed in external solution containing (in mM): NaCl 140, KCl 4, MgCl2 1, CaCl2 1, Glucose 10, HEPES 5 (pH 7.4, NaOH). The concentration of Iso used in the experiment was 10 nM. Access resistance was typically <10 MΩ after patch rupture. Action potentials were continuously elicited by square current pulses with an amplitude of 0.75-1 nA and a duration of 2-6 ms at stimulation rates of 0.5 and 1 Hz every minute at room temperature. Fast capacitance was compensated for in the cell-attached configuration. Recordings began after the cell had stabilized, which occurred approximately three minutes after rupture. The signals were filtered using 2.9- and 10-kHz Bessel filters and recorded using an EPC10 amplifier (HEKA Electronic). The recordings were analysed using LabChart 8.

### Electrophysiological recordings

Coverslips containing iPSC-CMs were transferred to a 37  ±  0.5 °C temperature-controlled chamber and superfused with bath solution containing (in mM): 4-aminopyridine 5 and BaCl₂ 0.1 (to reduce voltage-gated and inward rectifier K⁺ currents, respectively), as well as CaCl₂ 2, glucose 10, HEPES 10, KCl 4, MgCl₂ 1, NaCl 140, and probenecid 2 (pH 7.35 adjusted with NaOH). Membrane currents were recorded under voltage-clamp conditions using the whole-cell ruptured-patch configuration. Data acquisition and analysis were performed using pClamp software (version 10.7, Molecular Devices). The pipette solution contained (in mM): EGTA 0.02, GTP-Tris 0.1, HEPES 10, K-aspartate 92, KCl 48, Mg-ATP 1, and Na₂-ATP 4 (pH 7.2 adjusted with KOH). Tip resistances of borosilicate glass microelectrodes were 3–7 MΩ. I_Ca,L_ was measured at 0.5 Hz using a voltage-clamp protocol with a holding potential of −80 mV, consisting of a 100-ms ramp to −40 mV to inactivate the fast Na⁺ current (I_Na_), followed by a 100-ms depolarizing test pulse to +10 mV to activate L-type Ca²⁺ current, as previously described [1, 2]. Membrane capacitance was measured, and currents were expressed as current density (pA/pF).^40^

### Quantification of Arrhythmias

To quantify arrhythmic beating during Ca^2+^ measurements of atrial iPSC-CMs used in this study, we utilized the tool “*Arrhythpy*”.^43^ Briefly, the wavelet transformation (WT) was employed, providing both frequency and time information and yielding a two-dimensional coefficient map. Localized waves called wavelets (in this case Morlet wavelets) were used in the WT and the wavelet’s convolution was computed with the signal across various scales. The maxima and minima in the coefficient map highlight dominant frequencies at specific time points. By pairing each maximum with its corresponding minimum of the coefficient map, the width of a peak was estimated and converted into frequency values. The resulting time-resolved frequency profile was used to identify variance in frequencies (VF) indicating arrhythmias. The signal periodicity, calculated by the autocorrelation (AC), showed a normalised AC peak of one for rhythmic beating, while non-periodic signals had lower correlations. The peaks of the AC were averaged and inverted to yield a value between 0 and 1 (Inverted Mean Correlation; IMC), where 0 represents perfect periodicity and 1 indicates randomness. Combining VF and IMC into a single metric, called the arrhythmia measure (AM), provided a comprehensive assessment of arrhythmias.

### Phosphodiesterase activity

100,000 iPSC-CMs were cultured on Geltrex-coated cover slips (25 mm (R. Langenbrinck) and were transduced five days after digestion with exchange factor (EPAC) directly activated by cAMP 1 Cyclic adenosine monophosphate (EPAC1-cAMP) adenovirus (kindly provided by V. O. Nikolaev) with MOI 1 and 3. The measurements were performed 48 hours after transduction. The cells were washed and measured at room temperature in a buffer containing 5.4 mM KCl, 144 mM NaCl, 1 mM MgCl_2_, 10 mM HEPES and 2 mM CaCl_2_ (pH = 7.3). The following equipment was used: an inverted fluorescence microscope (IX71 Olympus), a polychrome V light source (Till Photonics), a beam splitter DV2 Dual-Channel (MAG Biosystems) and a charge-coupled device (CCD) camera iXon (Andor Technologies). The Förster Resonance Energy Transfer (FRET) donor CFP was excited at 440 nm and the exposure time was set to 60 - 100 ms. The CFP and YFP emissions were imaged every five seconds. The cells were treated with 100 nM Iso to induce catecholaminergic stress. After establishing baseline states, cells were treated with various Phosphodiesterase (PDE) inhibitors (100 nM Bay, 10 µM Cilostamid, 10 µM Roli, 10-minute incubation, respectively) and consecutively with 3-isobutyl-1-methylxanthine (IBMX) (100 µM, 15-minute incubation) to inhibit all PDEs. For additional testing of a PDE4 activator, MR-L2 (3 µM, 40-minute incubation) was applied to the TTS cells before measurement. The imaging data were analysed with MetaFluor 7.0 (Meta Imaging Series), Excel and GraphPad Prism9 software to calculate the FRET ratio corrected for bleed-through of the donor fluorescence CFP into the YFP acceptor channel (CFP/(YFP-0.7xCFP)).

### Western blot

Proteins in cell pellets (stored at −80 °C) were isolated for Western Blot Analysis. The cell pellet was resuspended in 100 µl Lysis Buffer, containing 20 mM Tris-HCl (pH = 7.4), 200 mM NaCl, 20 mM NaF, 1% Igepal, mM Na3VO4, 1 mM DTT, H_2_O and 1x PhosSTOP and 1x protease inhibitor. After one hour of incubation on ice, the solution was centrifuged at 3000 *g* for 8 minutes at 4 °C. The BCA (Bicinchoninic Acid)-Assay was performed to determine the protein concentration of each sample. 15 µg protein was mixed with 3.4 µl Laemmli Buffer, containing 90% Laemmli Buffer and 10% β-Mercaptoethanol. The samples were denatured for 30 minutes at 37 °C in a Thermocycler and separated by sodium dodecyl sulphate (SDS)-polyacrylamide gel electrophoresis (constant current of 20 mA/gel, run time 60–90 minutes). Protein transfer was performed with the Trans–Blot® TurboTM system chamber (transfer time 5 minutes, constant 25 V). After blocking with 5% milk for one hour at room temperature, the membrane was incubated on a shaker for 24 hours at 4° C with the primary antibody (PLN_Ser16p, dilution in 1% milk: 1:5000). After a washing process with 1 x TBST, the membrane was incubated at room temperature for one hour with the secondary antibody (Rabbit, IgG, dilution in 1% milk: 1:5000). After another washing step and subsequent incubation with horse radish peroxide, the membrane was imaged in an image maker (setting: Chemi high sensitivity). The band intensity was analysed with the Image Lab software.

### Whole Exome sequencing

Genomic DNA was quantified using Qubit fluorometry, and 200 ng was enriched with the SureSelect XT Human All Exon V5 kit before sequencing on an Illumina HiSeq2000 (2 × 75 bp). Demultiplexing and FASTQ generation were performed with bcl2fastq v1.8.4, and reads were aligned to hg19 using BWA. Duplicate reads were marked with Picard v1.64, and GATK v1.6 was applied for local realignment and quality score recalibration. Variant calling and filtering were conducted with the Varbank v2.3 pipeline (Cologne Centre for Genomics).

Variants were retained if coverage exceeded 6 reads, quality ≥10, and allele frequency ≥20%, and excluded if MAF ≥ 1% in public databases (dbSNP, 1000 Genomes, EVS, ExAC and gnomAD) or an in-house database. Pathogenicity was assessed with PROVEAN and SIFT, and variants were screened across 81 genes implicated in cardiomyopathy or arrhythmia. Both dominant (heterozygous) and recessive (compound heterozygous) models were considered.

### Statistical analysis

Statistical analysis was performed using the analysis function in GraphPad Prism9. For normally distributed data (Shapiro-Wilk normality test), the unpaired t-test was employed to assess the differences between two independent groups. For comparisons among more than two independent groups, an one-way analysis of variance (ANOVA) was performed. An ordinary two-way ANOVA with group and condition analysis was employed to explore the interaction between two independent variables. Differences in the proportion of cells exhibiting arrhythmic events were assessed using Fisher’s exact test. A two-sided *P*-value < 0.05 was considered statistically significant. Individual cells were treated as independent observations, as different cells were analysed under the respective conditions. Post hoc tests used the Tukey correction to account for multiple comparisons. When the assumptions of normality were not met, the non-parametric Mann-Whitney U test was used. To assess differences between more than two groups (non-normally distributed data), the Kruskal-Wallis test was used, followed by a Dunn post hoc test. All experimental data are presented as mean values ± Standard Error of the Mean (SEM).

## Supplementary information

Supplementary Material

Supplementary Figures

## Data Availability

All newly generated data from this study are publicly available.
