# Supplementary material for: Arrhythmic events and genetic variants as drivers for Takotsubo Syndrome: a patient-specific atrial stem-cell model
Source: Signal Transduct Target Ther. 2026 Oct 23;11:463. doi: 10.1038/s41392-026-03000-z (PMC13691113; doi:10.1038/s41392-026-03000-z)
Supplement: Supplementary file 1 [file 41392_2026_3000_MOESM1_ESM.docx]

**Supplementary Materials for**

**Arrhythmic events and genetic variants as drivers for Takotsubo Syndrome: a patient-specific atrial stem-cell model**

Charlotte Brand**^#^**, Clarissa Zinke**^#^**, Gideon Syed Ali, Daniela Hübscher, Karim Ajmail, Aylin Seedorf, Pauline Steinker, Franziska Treu, Philipp Jung, Hanna Eberl, Yun Li, Nataliya Dybkova, Lukas Feld, Jelena R. Ghadri, Christian Templin, Samuel Sossalla, Christoph Maack, Niels Voigt, Viacheslav O. Nikolaev, Bernd Wollnik, Katrin Streckfuss-Bömeke**^*^**

**#** These authors contributed equally (first authorship).

*** Corresponding author**

Prof. Dr. Katrin Streckfuss-Bömeke

Institute of Pharmacology and Toxicology

University of Würzburg, Germany

Versbacher Strasse 9, 97078 Würzburg

Tel: +49 931 31-84618

E-Mail: [katrin.streckfuss-boemeke@uni-wuerzburg.de](mailto:katrin.streckfuss-boemeke@uni-wuerzburg.de)

**This PDF file includes:**

Materials and Methods

Figures. S1 to S7

Tables S1 to S2

Materials and Methods

**Flow Cytometry**

The efficacy of each cardiac differentiation was probed for by flow cytometry of cardiac troponin T (cTNT) at day 60 of cardiac differentiation. The differentiation process was considered successful when more than 80 % of the cells exhibited positive cTNT expression. The CM monolayer was dissociated into single cells by incubation with 0.25 % Trypsin-EDTA for 10 minutes at 37 °C. The cells were then transferred to a flow tube (BD Falcon) containing fetal calf serum (FCS). This was followed by centrifugation at 200 g for 5 minutes and washing the cells twice with phosphate-buffered saline (PBS) (Thermo Fisher Scientific).

For fixation, the cells were incubated with 4 % Paraformaldehyde (ROTH) at room temperature for 20 minutes. After another centrifugation step and washing the cells twice with PBS, the samples were stored at 4 °C in a 1 % Bovine Serum Albumin (BSA) (Thermo Fisher Scientific) solution.

Cells were divided into three groups: blank, secondary antibody control, and cTNT sample. Cells were resuspended in FACS buffer (1 % BSA and 0,1 % Triton-X-100 (Sigma-Aldrich) in 1x PBS) and the cTNT sample was treated with the primary antibody (cTNT, 1:500, monoclonal mouse IgG (Thermo Fisher Scientific)) in FACS buffer and incubated overnight at 4 °C.

The following day, cTNT and control samples were washed three times with FACS buffer and incubated with the secondary antibody (Alexa Fluor 488 anti-mouse, 1:1000, donkey anti-mouse IgG (Life Technologies)) for 45 minutes at room temperature in the dark.

All samples were washed with 0.2 % BSA/PBS and resuspended in PBS for analysis on a FACS Canto II (BD Biosciences) (settings: 10,000 events, Forward Scatter 228 V, Side Scatter 440 V, and 488 Alexa Fluor Laser 390 V). The threshold for cTNT-positive cells was set using the blank sample.

**Immunostaining**

Approximately 20,000 CMs were cultured on Geltrex-coated cover slips (20 mm, (R. Langenbrinck)), fixed with 4 % Paraformaldehyde one week later for immunostaining and stored in 1 % BSA overnight at 4 °C. The cover slips were treated with the primary antibody in a 1 % BSA solution containing 0.1 % Triton X-100 and incubated overnight at 4 °C. The cells were incubated for one hour with the secondary antibody and subsequently with 4‘, 6-Diamidino-2-phenylindoldichloride (DAPI) (Sigma-Aldrich). The cover slips were mounted with Fluoromount-G (Southern Biotech) on microscope slides and imaged with a Zeiss Axio Observer.Z1.

The primary and secondary antibodies, the respective dilutions, host and supplier are listed in Supplementary Table 1.

**Quantitative real-time PCR (qPCR)**

The Biorad CFX Connect system was utilized for qPCR analysis. The reaction mixture was prepared by combining 5 ng/μl cDNA with 10 μl of SYBR green mix (2x), 1 μl of forward primer (10 μM), 1 μl of reverse primer (10 μM), and 8 μl of nuclease-free H_2_O (Thermo Fisher Scientific). This solution was then aliquoted into a well of a 96-well plate (Biorad). The cycling conditions were set as follows: Initial Denaturation: 3 minutes at 95 °C; Denaturation: 20 seconds at 95 °C; Primer Annealing: 20 seconds at 60 °C; Primer Elongation: 30 seconds at 72 °C; Additional Denaturation: 10 seconds at 95 °C; Melt Curve: 60 °C to 95 °C in 0.05 °C increments.

Steps 2-4 were repeated for a total of 40 cycles. The housekeeping gene GAPDH was used for relative mRNA quantification via the Δ-ct method. Positive and negative controls were included in the analysis alongside the samples. A Ct value greater than 35 was considered negative. The number of basepairs (bp), the primer sequences (forward and reverse), the annealing temperature, and the cycle number of each analysed gene are listed in Table S2.

**ALP staining**

Alkaline phosphatase (ALP), an enzyme highly active in stem cells, was detected using the Alkaline Phosphatase staining kit (Sigma-Aldrich). A 6-well plate containing iPSCs at 30-40 % confluency was washed with 1x PBS and fixed with the fixing solution (29 % citrate buffer (ddH_2_O, 2.94 g/l Sodium Citrate Dihydrate, 0.5 ml/l Tween-20 (adjusted to pH=6)), 65 % acetone, 6 % formaldehyde) for 30 seconds at room temperature. After washing the cells twice with dH_2_O, the cells were incubated with the staining solution (300 µl fast red violet (FRV) solution, 300 µl sodium nitrate, 13 ml dH_2_O, 30 µl Naphthol) at 37 °C for 20 minutes. After staining, the cells were air-dried overnight. Images of the ALP-stained colonies were captured using the AxiocamMR5 camera and Axio Rel 4.8 software (Zeiss).

**Spontaneous differentiation of iPSCs**

Inactivated mouse embryonic fibroblasts (MEFs), stored in liquid nitrogen, were thawed and resuspended in DMEM medium. After centrifugation for 5 minutes at 200g, the cells were resuspended in E8 medium. A total of 2.63 million MEFs and 5.25 million iPSCs were diluted in E8 medium (final volume: 20 ml). 200 µl of the cell suspension was pipetted into each well of a U-bottom shaped 96-well plate (Thermo Fisher Scientific), which was then centrifuged at 250 g for 5 minutes.On the following day and on every other day from then on, the medium was changed to Iscove differentiation medium (1x Iscove's Modified Dulbecco's Medium (IMDM), 20 % FCS, 1 % NEAA 100x (Thermo Fisher Scientific), 450 µM 1-Thioglycerine (MTG) (Sigma-Aldrich) (freshly added)). On day 8, eight embryoid bodies were transferred to each well of 12-well plates containing Geltrex-treated cover slides (20 mm). Each well received 1 ml of Iscove medium supplemented with MTG. Medium was changed every other day with 1.5 ml of Iscove + MTG medium. The cells were fixed on days 8, 10 and 8+25 for immunostainings. For visualization, the Zeiss Axio Observer.Z1 equipped with an Apotome was used. All used antibodies for immunofluorescence stainings can be found in Supplementary Table 1.

**Supplementary Fig. 1: Quality control of generated atrial iPSC-CMs.** Immunostaining of the sarcomere structure (α-Actinin = green, Titin = red, DAPI = blue) of a representative atrial iPSC-CM (scale bar: 20 µm) (**a**). 87.1 % ± 5.5 % of all differentiated atrial CMs are positive for cTNT (green dots) in flow cytometry (n = 52 cells/ 7 diffs) (Mann-Whitney U test) (scale bar: 2 µm) (**b**). Immunostainings of myosin light chain proteins (MLC2a = green, MLC2v = red, DAPI = blue) of atrial iPSC-CMs and ventricular iPSC-CMs (scale bar: 50 µm) (**c**). 90.9 % ± 6.4 % of the analysed cells are positive for MLC2a and negative for MLC2v (MLC2a+/MLC2v-; green dots), 7.6 % ± 5.6 % of cells are positive for both MLC2a and MLC2v (MLC2a+/MLC2v+; black dots) and 1.5 % ± 2.5 % of cells are negative for MLC2a and positive for MLC2v (MLC2a-/MLC2v+; red dots) (n = 29 diffs) (Kruskal-Wallis test with Dunn correction) (**d**). (e) and (f) atrial PC (=atrial differentiated CTRL iPSC-CM), atrial NC (ventricular differentiated CTRL iPSC-CM), ventricular PC (= ventricular differentiated CTRL iPSC-CM), ventricular NC (=atrial differentiated CTRL iPSC-CM). qPCR analysis for *NR2F2* expression (**e**) and *MLC2v* expression (**f**) (Kruskal-Wallis test with Dunn correction). Atrial negative control: n = 3 diffs (green dots); atrial CMs: n = 35 diffs (grey dots: CTRL, dark red dots: TTS, blue dots: CTRL-CASQ2, orange dots: CTRL-AHNAK, magenta dots: TTS-res-AHNAK); iPSC: n = 3 diffs (black dots); ventricular positive control: n = 3 diffs (red dots). All Data are presented as mean values ± SEM.

**Supplementary Fig. 2: a a**bsolute cAMP levels in EPAC1-based FRET measurements (CTRL (n= 44 cells/5 differentiations), TTS (n= 35/5)). Unpaired t-test. **b** EPAC1 biosensor-based FRET measurements of Rolipram sensitive part of PDE activity (PDE4) (as a percentage of the response to IBMX) on baseline level: CTRL (n= 16 cells/5 differentiations), TTS (n= 8/5), CTRL-CASQ2 (n= 16/5), CTRL-AHNAK (n= 16/5), TTS-res-AHNAK (n= 13/4). **c** Ca^2+^ transient amplitude on baseline level and after Rolipram (10 µmol/L) and Iso (10 nmol/L) treatment (CTRL: basal (n= 58/4), Roli (n= 41/4), Iso (n= 55/4), Roli+Iso (n= 54/4), TTS: basal (n= 49/4), Roli (n= 30/4), Iso (n= 45/4), Roli+Iso (n= 55/4). Two Way ANOVA with post hoc Tukey test. **d** 80 % relaxation time on baseline level and after Rolipram (10 µmol/L) and Iso (10 nmol/L) treatment (CTRL: basal (n= 58/4), Roli (n= 41/4), Iso (n= 55/4), Roli+Iso (n= 54/4), TTS: basal (n= 49/4), Roli (n= 30/4), Iso (n= 45/4), Roli+Iso (n= 55/4). Two Way ANOVA with post hoc Tukey test. **c+d** epifluorescence microscopy (Fura-2-AM). All Data are presented as mean values ± SEM.

**Supplementary Fig. 3.**

**Genetic analyses and characterisation of genome-edited cell lines.** Sanger Sequencing reveals *AHNAK1* mutation (p.5390T; c.16168C>A) in TTS and CTRL-AHNAK cell lines and a rescue of this mutation in TTS-res-AHNAK cells. CASQ2 mutation (p.F189L; c.567C>G) is found in TTS and CTRL-CASQ2 cells (**a**). Immunofluorescence analysis of CTRL-AHNAK and TTS-res-AHNAK iPSCs, confirming the expression of proteins of all three germ layers: α-smooth muscle actin (α-SMA) (green) for mesoderm, α-fetoprotein (AFP) (red) for endoderm, and β-III-tubulin (green) for ectoderm (blue = DAPI) (scale bar: 100µm) (**b**). CTRL-AHNAK (above) and TTS-res-AHNAK (below): Morphology image showing a colony of characteristically shaped iPSCs (scale bar: 100 µm); positive ALP (red) staining (scale bar: 100µm); positive staining of standard pluripotency markers, including OCT4, SOX2, LIN28, TRA1-60 and NANOG (scale bars: 50 µm) (**c**).

**Supplementary Fig. 4.**

**Karyotype analysis of CTRL-AHNAK.** No chromosomal abnormalities were found in CTRL-AHNAK cells.

**Supplementary Fig. 5.**

**Off target analysis of CTRL-AHNAK and TTS-res-AHNAK.** The top five potential off-target sites in the genome-edited cell lines were identified using the Jefferson "Off-spotter" tool. For the CTRL-AHNAK cell line off-target analysis includes the genes: apoptosis inhibitor 5 (*API5*), tuftelin 1 (*TUFT1*), endothelin converting enzyme 1 (*ECE1*), Chromosome 12 Open Reading Frame 42 (*C12orf42)*, Bassoon Presynaptic Cytomatrix Protein (*BSN*) (**a**). For the TTS-res-AHNAK cell line the sequenced off-target genes included: Taxilin Gamma *(TXLNG),* F-Box and Leucine Rich Repeat Protein 7 *(FBXL7),* JAZF Zinc Finger 1 *(JAZF1),* NKD Inhibitor of WNT Signaling Pathway 1 *(NKD1),* TATA-Box Binding Protein Associated Factor 9 *(TAF9)* (**b**).

**Supplementary Fig. 6.**

***CASQ2* and *AHNAK* expression on mRNA level.** qPCR analysis of CASQ2 gene expression (CTRL (n = 4), TTS (n = 3), CTRL-CASQ2 (n = 5), CTRL-AHNAK (n = 5), TTS-res-AHNAK (n = 4) (**a**). qPCR analysis of *AHNAK1* gene expression (CTRL (n = 4), TTS (n = 3), CTRL-CASQ2 (n = 5), CTRL-AHNAK (n = 5), TTS-res-AHNAK (n = 4) (**b**) One Way Anova with Post hoc Tukey test. Additionally, exploratory pairwise comparisons using unpaired t tests were performed to further investigate specific group differences (TTS vs. TTS-res-AHNAK). All Data are presented as mean values ± SEM.

**Supplementary Fig. 7.**

**Protein sequence conservation between species.** Protein sequence conservation of the amino acids P5390 in AHNAK and F189 in CASQ2 between species.

Supplementary Table 1.

**Overview of primary and secondary antibodies for immunofluorescence staining with respective dilution, host and supplier.**

| **Primary antibody** | **Dilution in 1 % BSA** | **Type** | **Supplier** |
| --- | --- | --- | --- |
| α-Actinin | 1:500 | Monoclonal mouse IgG_1_ | Sigma-Aldrich |
| α-fetoprotein (AFP) | 1:500 | Polyclonal rabbit IgG | DAKO |
| α-SMA | 1:3000 | Monoclonal mouse IgG_2A_ | Sigma-Aldrich |
| β-III-Tubulin | 1:2000 | Monoclonal mouse IgG_2A_ | Covance |
| LIN28 | 1:300 | Polyclonal goat IgG | R&D Systems |
| Myosin light chain atrium (MLC2a) | 1:200 | Monoclonal mouse IgG_2B_ | Synaptic Systems |
| Myosin light chain ventricular (MLC2v) | 1:200 | Polyclonal rabbit IgG | Proteintech |
| NANOG | 1:50 | Polyclonal goat IgG | R&D Systems |
| OCT4 | 1:40 | Polyclonal goat IgG | R&D Systems |
| SOX2 | 1:200 | Monoclonal mouse IgG_2A_ | R&D Systems |
| Titin M-line M8/M9 | 1:750 | Polyclonal rabbit IgG | Myomedix |
| TRA-1-60 | 1:200 | Monoclonal mouse IgM | Abcam |
| **Secondary Antibody** | **Dilution in 1 % BSA** | **Type** | **Supplier** |
| Alexa Fluor 488 anti-mouse | 1:1000 | Donkey anti-mouse IgG | Life Technologies |
| Alexa Fluor 488 anti-mouse | 1:200 | Goat anti-mouse IgG+IgM | Life Technologies |
| Alexa Fluor 555 anti-goat | 1:1000 | Donkey anti-goat IgG | Life Technologies |
| Alexa Fluor 555 anti-rabbit | 1:1000 | Donkey anti-rabbit IgG | Life Technologies |
| Cy3 anti-mouse | 1:300 | Goat anti-mouse IgG+IgM | Jackson ImmunoResearch Laboratories |

Supplementary Table 2.

**Overview of the product size, the forward and reverse primer sequence, the annealing temperature, and the cycle number of the respective genes.**

| **Gene name** | **Product size (bp)** | **Forward primer sequence** | **Reverse primer sequence** | **Annealing temperature** | **Cycle** |
| --- | --- | --- | --- | --- | --- |
| *AHNAK* | 170 | CCAGATGTGACACTGAGGGG | CCCGGAGTAGAGATGCCAAA | 60° C | 40 |
| *CASQ2* | 131 | GCAGCAAACTGGAAGTCCAAGC | GATGTAAGGCTGGAAGTGTTTC | 60° C | 40 |
| *GAPDH* | 110 | GTCTCCTCTGACTTCAACAGCG | ACCACCCTGTTGCTGTAGCCAA | 60° C | 40 |
| *MLC2v* | 200 | GGCGAGTGAACGTGAAAAAT | CAGCATTTCCCGAACGTAAT | 60° C | 40 |
| *NR2F2* | 226 | CCGACCGGGTGGTCGCCTTTATGGA | CGGCTGGTTGGGGTACTGGCTCCTA | 60° C | 40 |
